# The *Chlamydia pneumoniae* Invasin Protein Pmp21 Recruits the EGF Receptor for Host Cell Entry

**DOI:** 10.1371/journal.ppat.1003325

**Published:** 2013-04-25

**Authors:** Katja Mölleken, Elisabeth Becker, Johannes H. Hegemann

**Affiliations:** Funktionelle Genomforschung der Mikroorganismen, Heinrich-Heine Universität, Düsseldorf, Germany; Collège de France, France

## Abstract

Infection of mammalian cells by the strictly intracellular pathogens *Chlamydiae* requires adhesion and internalization of the infectious Elementary Bodies (EBs). The components of the latter step were unknown. Here, we identify *Chlamydia pneumoniae* Pmp21 as an invasin and EGFR as its receptor. Modulation of EGFR surface expression evokes correlated changes in EB adhesion, internalization and infectivity. Ectopic expression of EGFR in EGFR-negative hamster cells leads to binding of Pmp21 beads and EBs, thus boosting the infection. EB/Pmp21 binding and invasion of epithelial cells results in activation of EGFR, recruitment of adaptors Grb2 and c-Cbl and activation of ERK1/2, while inhibition of EGFR or MEK kinase activity abrogates EB entry, but not attachment. Binding of Grb2 and c-Cbl by EGFR is essential for infection. This is the first report of an invasin-receptor interaction involved in host-cell invasion by any chlamydial species.

## Author Summary

The obligate intracellular bacterial pathogen *Chlamydia pneumoniae* is an important cause of human and animal diseases and can infect various cell types. The molecular mechanisms of chlamydial adhesion to and invasion of human cells are not well defined. Recently we identified Pmp21 and other members of the family of polymorphic membrane proteins (Pmp) as the first chlamydial adhesins binding to proteinaceous host cell-surface structures. Here we show that recombinant Pmp21 functions as an invasin protein. Using a biochemical approach we identified the human epidermal growth factor receptor (EGFR) — an ubiquitously expressed cell surface-localized receptor tyrosine kinase — as the cellular receptor for Pmp21, making Pmp21 the first pathogen-derived EGFR ligand. The EGF receptor is recruited to adherent and internalized EBs. Depletion of EGFR from the human cell surface significantly reduced chlamydia adhesion and internalization. Likewise, ectopic expression of EGFR in receptor-negative cells increased chlamydia adhesion, internalization and subsequent infectivity. Binding of Pmp21 to EGFR initiates receptor activation and downstream signaling, both of which we found to be equally important for bacteria entry. In conclusion, we show that the Pmp21 adhesin binds and activates EGFR, which initiates signaling cascades, finally leading to chlamydia/receptor internalization.

## Introduction

The genus *Chlamydia* comprises obligate intracellular, Gram-negative pathogens that infect a variety of organisms. The *Chlamydia pneumoniae* infection is ubiquitous in humans, with an antibody prevalence of 50% by age 20 years. *C. pneumoniae* is a common cause of community-acquired pneumonia and other respiratory infections. Moreover, its persistent infection may play a role in chronic inflammation and atherosclerosis [1]. All *Chlamydia* species share a common biphasic developmental cycle, characterized by adhesion and internalization of infectious, metabolically inactive elementary bodies (EBs) into a membrane-bounded compartment, termed inclusion. How the bacteria are internalized by host cells is largely unknown.

After attachment of *Chlamydiae* to host cells subsequent internalization may occur either by clathrin-mediated endocytosis or via caveolin-rich domains or lipid rafts [2], [3], [4]. Activation of specific signaling pathways upon attachment, and subsequent rearrangement of actin networks, are essential for entry [5], [6]. Infection by *C. pneumoniae* is associated with activation of tyrosine kinases, PI3-dependent and MAP kinases, and leads within minutes to activation of ERK via the Ras-Raf-MEK cascade [6], [7]. The focal adhesion kinase (FAK) is tyrosine phosphorylated within minutes of exposure to *C. pneumoniae*, and may recruit additional signaling molecules to sites of bacterial attachment [6]. Isoform-specific tyrosine phosphorylation of the docking protein SHC also occurs at the time of *C. pneumoniae* attachment and entry suggesting activation of yet unknown receptors [6].

Since *Chlamydia* species can infect different cell types *in vitro*, they may use widespread host-cell receptors and/or a broad repertoire of specific chlamydial adhesins. The *C. pneumoniae* Pmp6, Pmp20 and Pmp21 proteins are recently identified adhesins essential for EB adhesion to human cells [8]. However, the receptor(s) for these adhesins remain(s) unknown.

In this study, we demonstrate that Pmp21 acts as an invasin protein for *C. pneumoniae* and identify the epidermal growth factor receptor (EGFR) as its direct interaction partner. The interaction leads to activation of EGFR. Furthermore, we show that the activated receptor is tightly associated with internalized Pmp21-coated beads and is also clustered in ring-like structures around the internalized *C. pneumoniae* EBs. Expression of functional EGFR on human cells is essential for binding and internalization of the bacteria. Finally, recruitment of the adaptor proteins Grb2 and c-Cbl by EGFR is essential for infection by *C. pneumoniae*. The Pmp21-EGFR interaction thus represents the missing link between chlamydial attachment and the subsequent host cell entry.

## Results

### Pmp21-coated latex beads are taken up by mammalian cells

**Figure 1:**
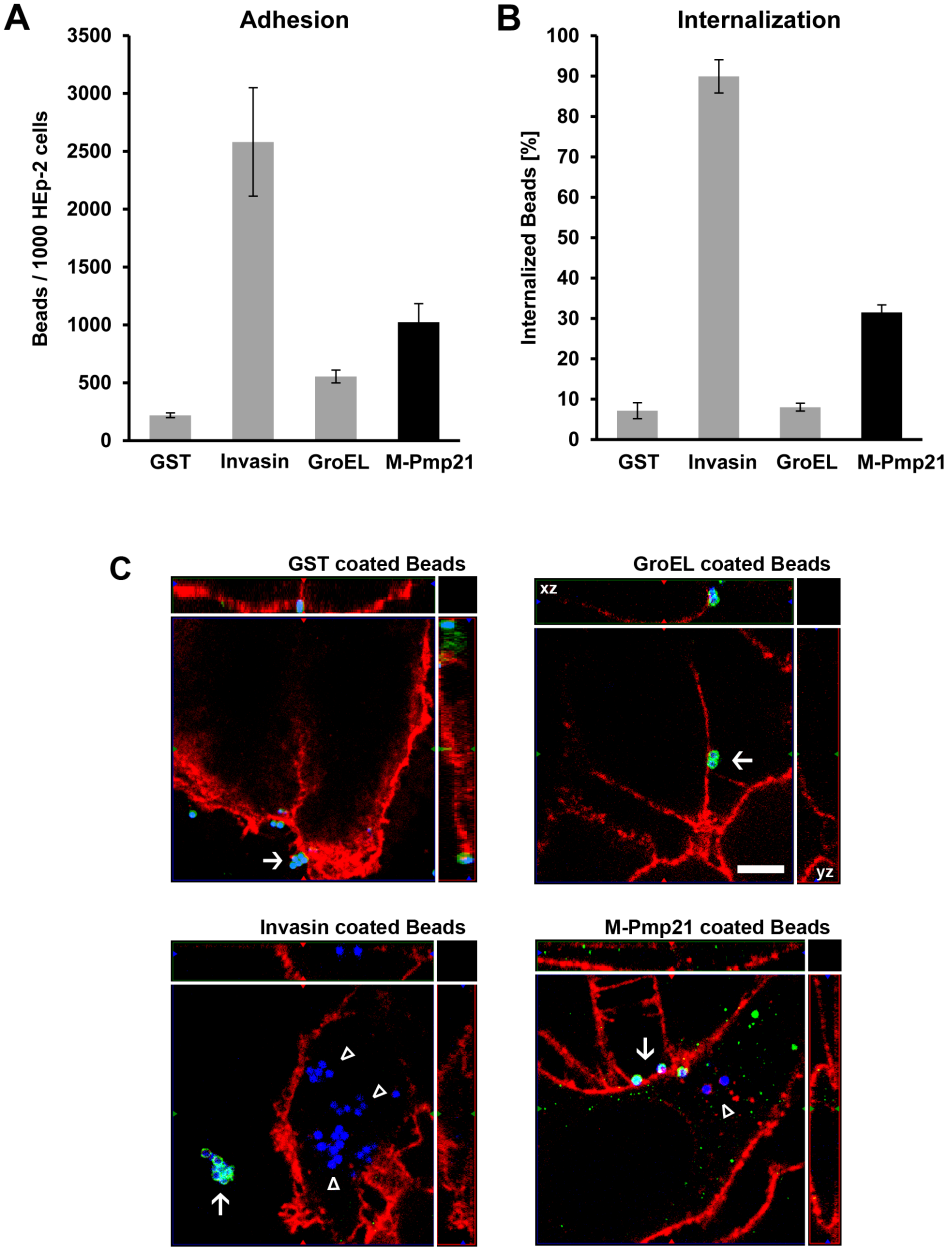
Pmp21-coated beads are taken up by mammalian cells. (**A**) Green fluorescent beads coated with recombinant GST, invasin, GroEL1 or M-Pmp21 were incubated in 5-fold excess with HEp-2 cells for 1 h at 4°C, and the numbers of beads found on 1000 HEp-2 cells were counted (n = 3). (**B**) Internalization of M-Pmp21-coated beads. HEp-2 cells were incubated with beads as above at 37°C for 4 h, and washed with PBS to remove unattached beads. Attached beads were stained with specific antibodies without cell permeabilization (see Figure S1), and the numbers of attached (red) and internalized (green) beads on/in samples of 1000 cells were counted (n = 3). (**C**) Confocal spinning-disk images of internalized beads (for the GroEL image the xz and yz plain projections of the whole field are marked). HEp-2 cells were incubated for 4 h at 37°C with blue fluorescent beads coated with GST, invasin, GroEL1 or M-Pmp21. External beads were stained with protein specific antibodies (green). Internalized beads are not accessible to the antibody and appear blue. Cell boundaries were stained with Wheat Germ Agglutinin-Alexa594 (red). External beads are marked by white arrows, internalized beads by white triangles. Bar 5 µm.

The recent identification of Pmp21 as a *C. pneumoniae* adhesin led us to ask whether it can be internalized by host cells. Infectious EBs bear proteolytically processed forms of Pmp21 on their surfaces (summarized in [8], [9], [10]). N-Pmp21, M-Pmp21 and N/M-Pmp21 all mediate adhesion of EBs to human epithelial (HEp-2) cells and (in soluble form) block infection by *C. pneumoniae* [8]. We incubated microbeads coated with one of four recombinant (His-tagged) proteins with HEp-2 cells at 4°C. Beads loaded with tagged GST showed little binding, while beads bearing recombinant invasin from *Yersinia pseudotuberculosis*, GroEL1 from *C. pneumoniae* or M-Pmp21 clearly bound to the cells (Figs. 1A, S1). After further incubation at 37°C, 7% of GST and of GroEL1 beads respectively, were found in cells (Figs. 1B, S1), while 90% of adherent invasin-coated beads and 31% of Pmp21-coated beads were internalized (Figs. 1B, 1C and S1). Hence M-Pmp21 induces bead uptake into these epithelial cells via a specific receptor.

### Identification of the EGF receptor as an interaction partner for Pmp21

**Figure 2:**
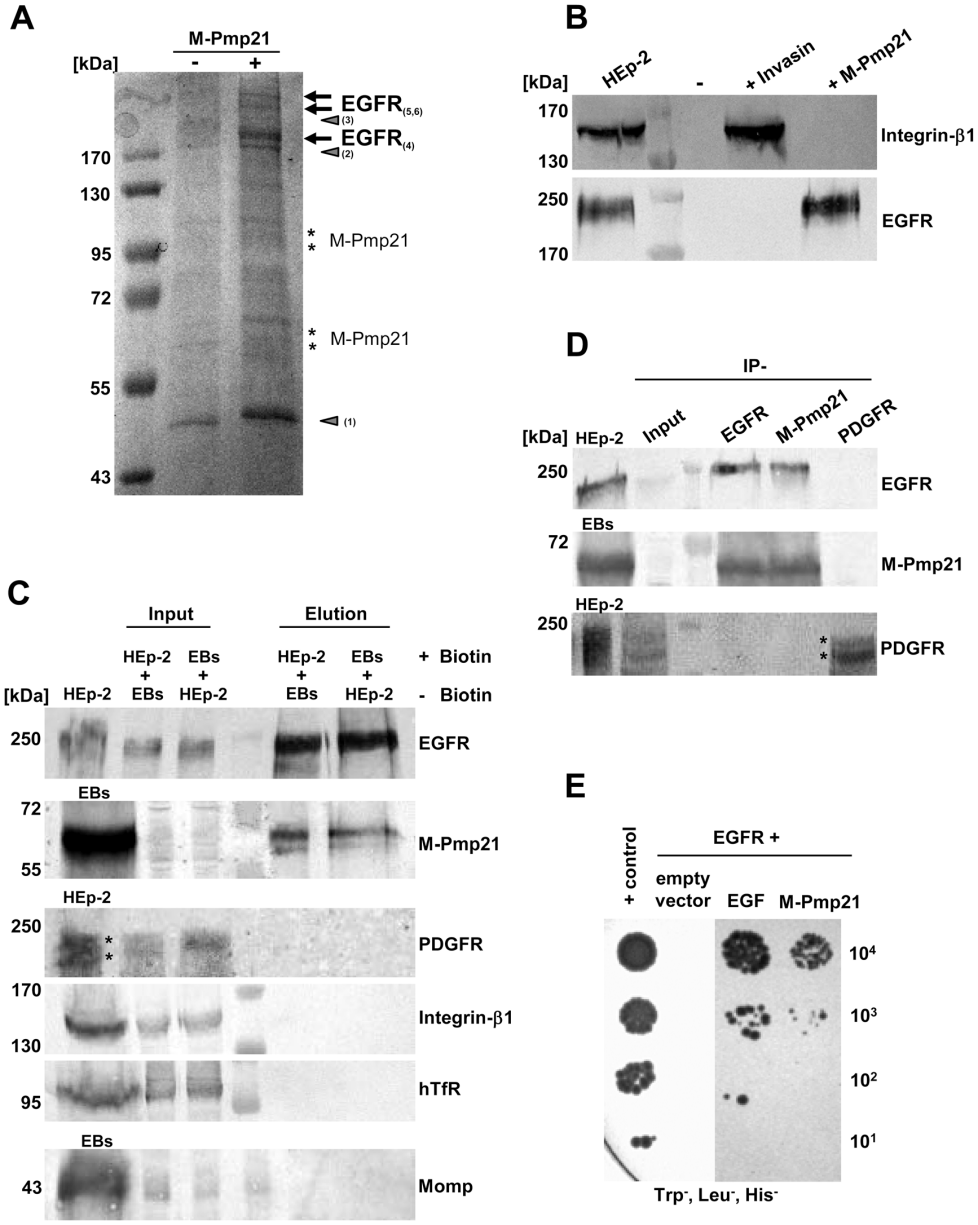
Identification of the EGF receptor as an interaction partner for the *C. pneumoniae* adhesin Pmp21. (**A**) Electrophoretic analysis of fractions eluted from a NeutrAvidin column. HEp-2 cells were incubated with (+) or without (−) biotinylated M-Pmp21 or invasin (data for invasin not shown), and processed as described in Experimental Procedures. Arrows mark bands in which EGFR was identified by MALDI-MS (see Fig. S2B; bands 4–6). Other major bands (triangles) were identified as actin (1) and pleckstrin (2); no protein was detected in band 3. Bands marked with asterisks represent the recombinant M-Pmp21 as shown by immunoblotting (data not shown). (**B**) Fractions from (**A**) were probed with anti-integrin-β1 and anti-EGFR antibodies. (**C**) Affinity purification of the EGFR-Pmp21 complex from surface-biotinylated HEp-2 cells incubated with non-biotinylated *C. pneumoniae* EBs, or *vice versa*, for 60 min at 37°C. After crosslinking the biotinylated surface protein complexes were bound to a NeutrAvidin column. After crosslink removal the eluted proteins were identified by immunoblot analysis with specific antibodies against EGFR, M-Pmp21, PDGFR, integrin-β1, hTfR and Momp. Equal amounts of input and elution samples were loaded onto the SDS-PAGE. (**D**) Coimmunoprecipitation of the EGFR-Pmp21 complex from HEp-2 monolayers incubated with purified *C. pneumoniae* EBs (MOI 5) for 60 min. The Pmp21-EGFR complex was precipitated using EGFR-, M-Pmp21- and PDGFR-specific antibodies as indicated, and probed after SDS-PAGE and immunoblotting. Stars mark the 2 main PDGFR protein species recognized. (**E**) Interaction of EGFR and M-Pmp21 shown by Y2H analysis. Serial dilutions (10^1^–10^4^) of yeast cells expressing EGFR/EGF or EGFR/M-Pmp21 were patched on low-stringency selection medium (Leu^−^, Trp^−^, His^−^). + control: SV40 LTA/p53.

We chose a biochemical approach to identify the host receptor for Pmp21. rM-Pmp21 labeled with NHS-SS-biotin was incubated with a monolayer of HEp-2 cells to allow it to interact with its cellular target(s) (Fig. S2A). Bound M-Pmp21 was then cross-linked to its partner(s) on the cell surface using the membrane-impermeable reagent DTSSP. The cells were then lysed, and the lysates were applied to a NeutrAvidin column to capture biotinylated complexes (see Experimental Procedures). Biotin-bound protein complexes were eluted, and crosslinks simultaneously cleaved, with DTT, and eluted fractions were subjected to SDS/PAGE. As a control, an identical set-up was used with invasin as the probe (data not shown). Several bands not found in the control lanes (no M-Pmp21 added) were analyzed by MALDI-MS and peptide mass fingerprinting (Figs. 2A, S2B). Three bands (>170 kDa) detected only in lysates of HEp-2 cells exposed to biotinylated M-Pmp21 were identified as the human epidermal growth factor receptor (EGFR/HER1 or ErbB-1). An anti-EGFR antibody confirmed the presence of EGFR in the rM-Pmp21 lysate and its absence in the control invasin lysate. Conversely, the invasin receptor integrin-β1 was detectable only in the latter lysate (Fig. 2B).

To confirm the interaction of Pmp21 with EGFR *in vivo*, the crosslinking/affinity purification procedure was applied to cell lysates after surface-biotinylated EBs had been incubated with non-biotinylated HEp-2 cells or *vice versa*, and immunoblots were probed with anti-EGFR or anti-Pmp21 antibodies (Fig. 2C). When biotinylated EBs or biotinylated HEp-2 cells were used in this test, the EGFR and the Pmp21 signals were strongly increased in the elution fractions compared to input. Probing of eluates with antibodies against PDGFR, integrin-β1, human transferrin receptor, the tyrosine receptor kinase Met (data not shown) or the bacterial cell surface protein Momp gave no signals, indicating that the EGFR-Pmp21 interaction is specific (Fig. 2C).

Immunoprecipitation experiments on infected cells confirmed these data. HEp-2 cells exposed to *C. pneumoniae* for 1 h were crosslinked, and membrane protein complexes were solubilized and immunoprecipitated with EGFR-, PDGFR- or M-Pmp21-specific antibodies. A specific interaction was again detected between M-Pmp21 and EGFR, but not between M-Pmp21 and PDGFR (Fig. 2D). Thus, affinity labeling and immunoprecipitation experiments strongly suggest that Pmp21 interacts specifically with EGFR.

To show that this interaction is direct, yeast two-hybrid (Y2H) analyses were performed (Figs. 2E, S4A). Human EGFR (aa 1 – aa 1209; see [11]) was expressed as a fusion to the Gal4 activation domain and tested for interaction with its natural ligand EGF, or with M-Pmp21, each fused to the Gal4 binding domain. Patch tests revealed that only yeast cells that co-expressed EGFR with either EGF or M-Pmp21 could grow on selective medium, indicating that M-Pmp21 physically interacts with EGFR, but not with other receptors (e.g. the LDL receptor) in this surrogate system (Figs. 2E, S4A+B).

### EGFR colocalizes with chlamydial EBs during the infection process

**Figure 3:**
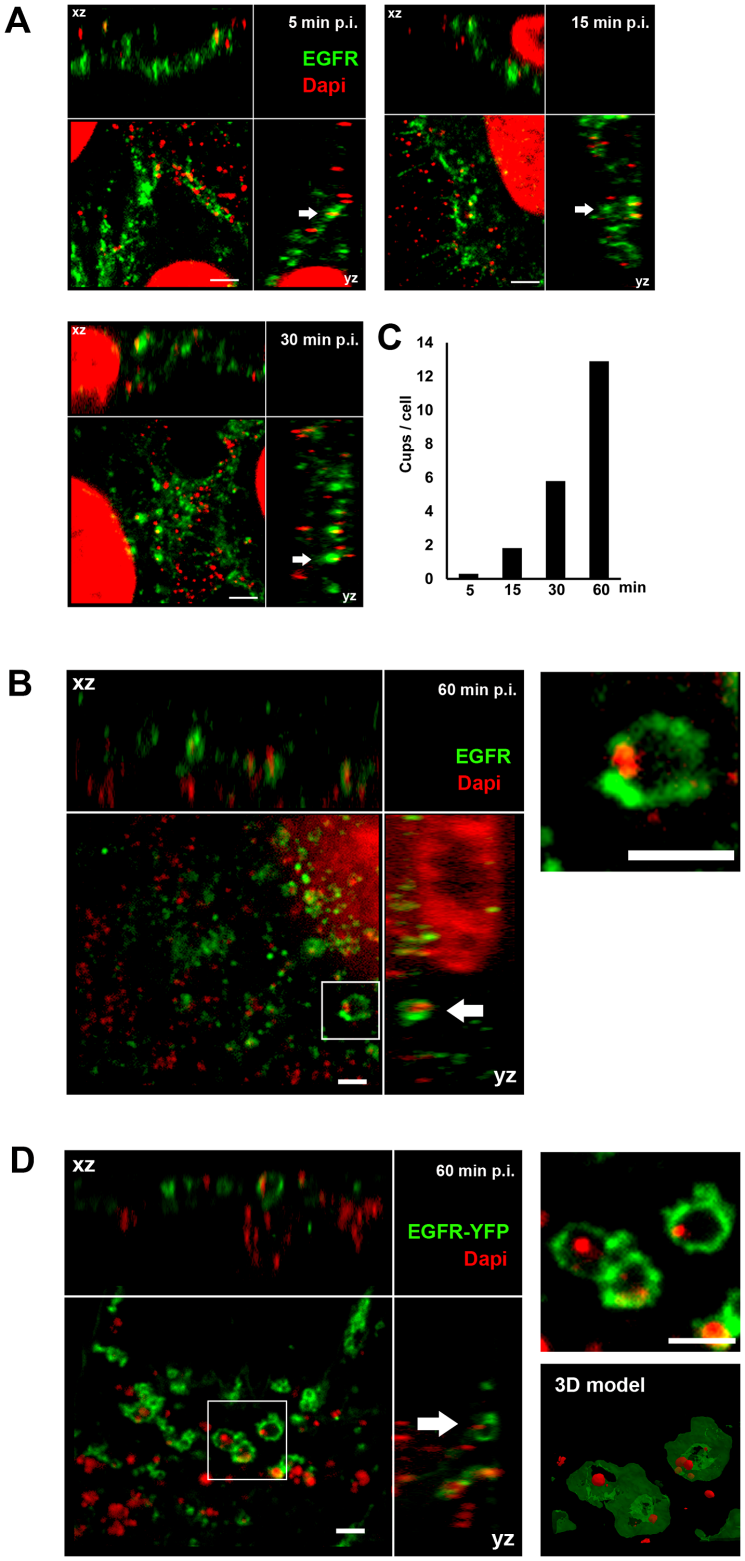
EGFR colocalizes with EBs at bacterial entry sites. (**A–B**) Confocal spinning-disk immunofluorescence microscopy of HEp-2 cells exposed to *C. pneumoniae* EBs for 5 min, 15 min, 30 min (**A**) or 60 min (**B**). Fixed cells were stained with anti-EGFR (green) and EBs with DAPI (red). Arrows indicate colocalization of the two signals. (**B**) *C. pneumoniae* EBs surrounded by ring-like EGFR structures 60 min pi. Arrows indicate colocalization of EGFR with DAPI-stained EBs at bacterial entry sites in xz and yz planes. An internalized EB surrounded by EGFR (white box) is shown at higher magnification on the right. (**C**) Quantification of colocalization events (cups) of EBs and EGFR signals at the indicated time points. The data are from a typical experiment. (**D**) Transfected CHO-K1 cells expressing human EGFR tagged with YFP (green) reveal colocalization of EGFR with DAPI-stained EBs (red) at bacterial entry sites 60 min pi. An internalized EB surrounded by EGFR-YFP (white box) is shown at a higher magnification on the upper right, and a 3D model of the structure (based on a Maximal Intensity Projection) is shown below it (see movie S1). Bar 1 µm.

Since EGFR on the host-cell surface interacts with the EB-associated Pmp21 early in infection, we followed its later relationship with *C. pneumoniae* EBs by indirect immunofluorescence microscopy. At 5 min and 15 min pi, between 0.3 and 1.8 clustered EGFR signals (termed cups) were found to be associated with *C. pneumoniae* EBs attached to single cells, and this increased to about 6 EGFR cups per cell by 30 min pi (Figs. 3A, 3C). At 60 min pi EBs were frequently colocalized with the EGFR, which formed cups or ring-like structures around internalized bacteria, often near the nucleus (Figs. 3B, 3C). Similarly, in CHO-K1 cells expressing EGFR-YFP, recruitment of the receptor to rings surrounding chlamydial particles was detectable (Fig. 3D). A 3D model of the structures seen at 60 min pi revealed the clustering of the receptor (Fig. 3D; see also movie S1). The morphology of these ring-like forms implies that the EGFR is recruited to the membrane around the EBs as they are endocytosed and remains associated with the early inclusion thus formed.

### Depletion of EGFR from the cell surface inhibits infection

**Figure 4:**
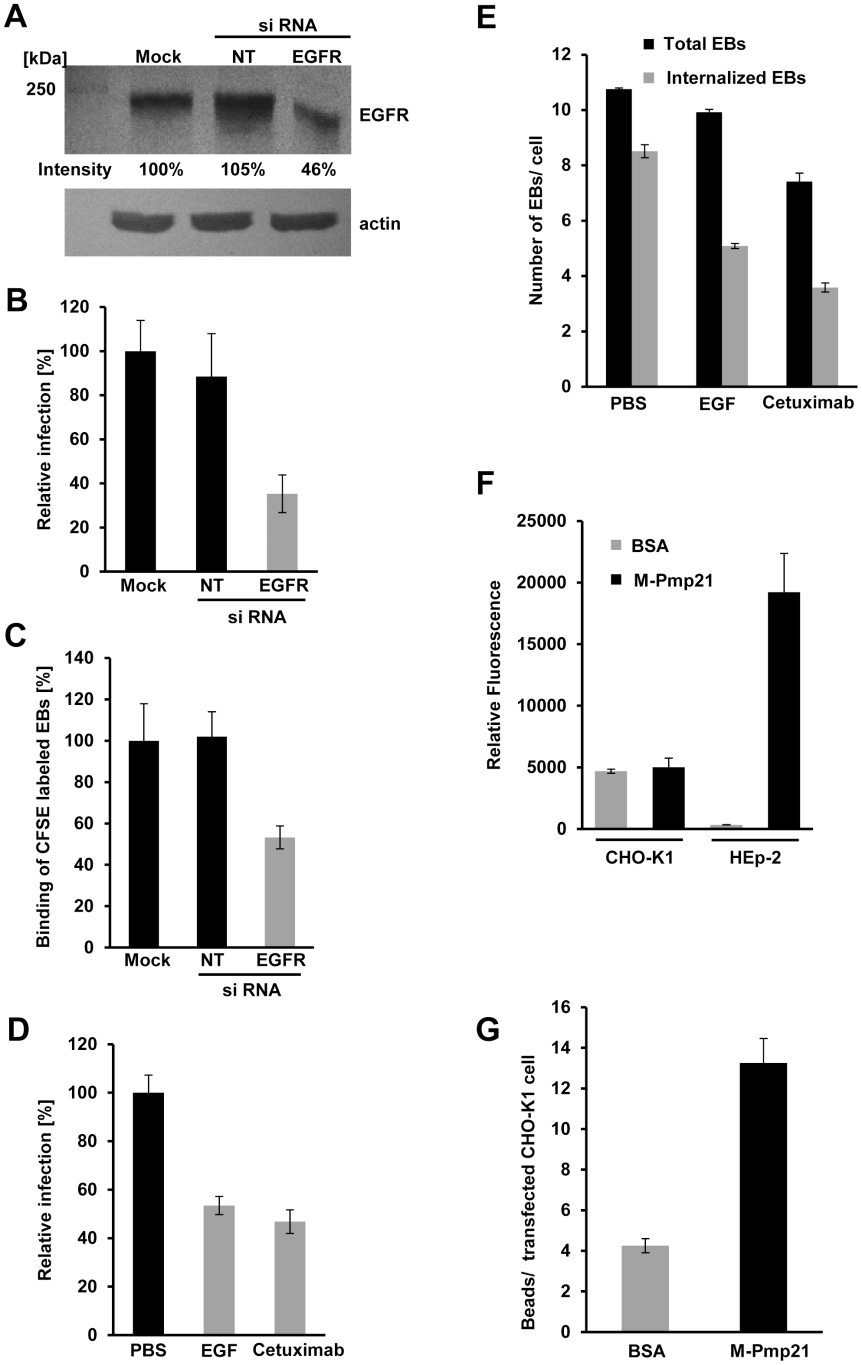
EGFR is required for internalization of *C. pneumoniae*. (**A–C**) HeLa229 cells were mock-transfected (Mock), or transfected with a non-targeting siRNA (NT) or an EGFR-targeting siRNA for 24 h. (**A**) Relative EGFR levels were determined using Scion Image software after immunoblot analysis with anti-EGFR. Actin served as loading control. (**B**) HeLa229 cells transfected for 24 h were infected with *C. pneumoniae* GiD (MOI 1) for 48 h. Inclusion formation was evaluated by indirect immunofluorescence with an FITC-conjugated antibody against chlamydial LPS. The number of inclusions in mock-transfected cells was set to 100% (n = 4). (**C**) *C. pneumoniae* EBs labeled with CFSE were added (MOI 10) to transfected HeLa229 cells for 1 h at 37°C. Cells were then detached from the substrate and fixed with formaldehyde, and adhesion was measured by flow cytometry. The mean fluorescence of EBs bound to mock-transfected cells was set to 100% (n = 3). (**D**) Pretreatment of confluent HEp-2 monolayers with recombinant EGF or Cetuximab for 2 h inhibits subsequent infection (n = 4) (MOI 1). (**E**) Pretreatment of HEp-2 cells with rEGF or Cetuximab for 2 h inhibits subsequent internalization of *C. pneumoniae* EBs (MOI 1). Cells were exposed to EBs for 2 h, then fixed and stained with anti-Pmp21 and DAPI without permeabilization. Numbers of internalized EBs (inaccessible to anti-Pmp21 antibody) were determined by subtracting the number of external EBs (visualized with anti-Pmp21) from the total number of EBs (DAPI stain) in each cell (n = 5). (**F**) rM-Pmp21-coated beads adhere to HEp-2 but not CHO-K1 cells. A five-fold excess of green fluorescent beads coupled to BSA or rM-Pmp21 were incubated with CHO-K1 and HEp-2 cells for 1 h at 37°C. Unbound beads were removed by washing with PBS, and cells bearing attached beads were analyzed by flow cytometry. The mean fluorescence values for the samples analyzed (n = 4) are shown. (**G**) rM-Pmp21-coated beads attach to EGFR-expressing CHO-K1 cells. CHO-K1 cells were transfected with EGFR-mCherry for 24 h, incubated with a five-fold excess of green fluorescent beads coated with BSA or rM-Pmp21, and the numbers of beads attached to transfected cells were determined (n = 3).

The EGFR/Pmp21 interaction data and colocalization of EGFR with EBs in endocytic vesicles imply an important function for EGFR in the initiation of infection. To further study this role, the level of the protein was down-regulated using specific siRNA in epithelial HeLa229 cells, which could be transfected efficiently. By 24 h after transfection, EGFR levels were estimated to be less than half those in control cells (Fig. 4A) and the infectivity (based on the number of inclusions at 48 h pi) of *C. pneumoniae* was reduced by 64% compared to the mock-transfected control (Fig. 4B). Binding of labeled *C. pneumoniae* EBs to transfected cells was found to be reduced by some 50% (Fig. 4C). Hence the expression level of EGFR correlates with the level of EB attachment and subsequent infection, indicating that the receptor is involved in mediating infection.

Preincubation of human cells with EGF transiently removes EGFR from the cell surface by inducing EGFR signaling and internalization of the receptor-ligand complex [12]. When cells were incubated with EGF for 2 h, and then exposed to *C. pneumoniae*, infectivity was reduced by 47% compared to the PBS control (Fig. 4D). Interestingly, the number of EBs associated with the cells fell by only about 7% compared to the control, while the number of internalized chlamydial cells was reduced by 42% (Fig. 4E). Thus, after exposure of cells to EGF, sufficient EGFR remains on the surface to bind most of the EBs on offer.

To confirm these results, HEp-2 cells were treated for 2 h with the antibody cetuximab, which blocks the ligand-binding site of EGFR [13] and simultaneously triggers receptor endocytosis, thus depleting EGFR from the cell surface [14]. Treatment of cells with the antibody prior to exposure to *C. pneumoniae* reduced infectivity by 54% compared to control (Fig. 4D). The total number of EBs associated with the HEp-2 cells fell by only 32%, but a 60% reduction in internalization of the infectious EBs was observed (Fig. 4E). These data are all consistent with the idea that Pmp21 mediates binding of EBs to EGFR. To prove this directly we tested whether rM-Pmp21-coated beads could bind to CHO-K1 cells, which lack EGFR [15]. Indeed CHO-K1 cells bind Pmp21-bearing beads no more efficiently than BSA control beads, while HEp-2 cells, which express high levels of EGFR showed very significant M-Pmp21 bead binding (Fig. 4F). In contrast, more than three times as many M-Pmp21 beads as BSA control beads bound to CHO-K1 cells transfected with EGFR (Fig. 4G). These data directly demonstrate that the Pmp21 adhesin binds to EGFR.

### Both *C. pneumoniae* EBs and the adhesin Pmp21 recruit and activate EGFR

EGFR belongs to the family of receptor tyrosine kinases [16], [17]. It is activated by several natural ligands, but direct binding of a microbial pathogen by this receptor had not been described hitherto [18]. We therefore asked whether EGFR is activated upon binding of Pmp21 and chlamydial EBs. Binding of EGF to EGFR leads to dimerization of the receptor, activation of its intrinsic kinase function and autophosphorylation of critical tyrosine residues located in the C-terminal tail facing the cytosol [19], [20]. The phosphorylated tyrosines provide an interaction platform for cytosolic proteins involved in endocytosis of the activated receptor and for members of downstream signaling cascades [21], [22].

**Figure 5:**
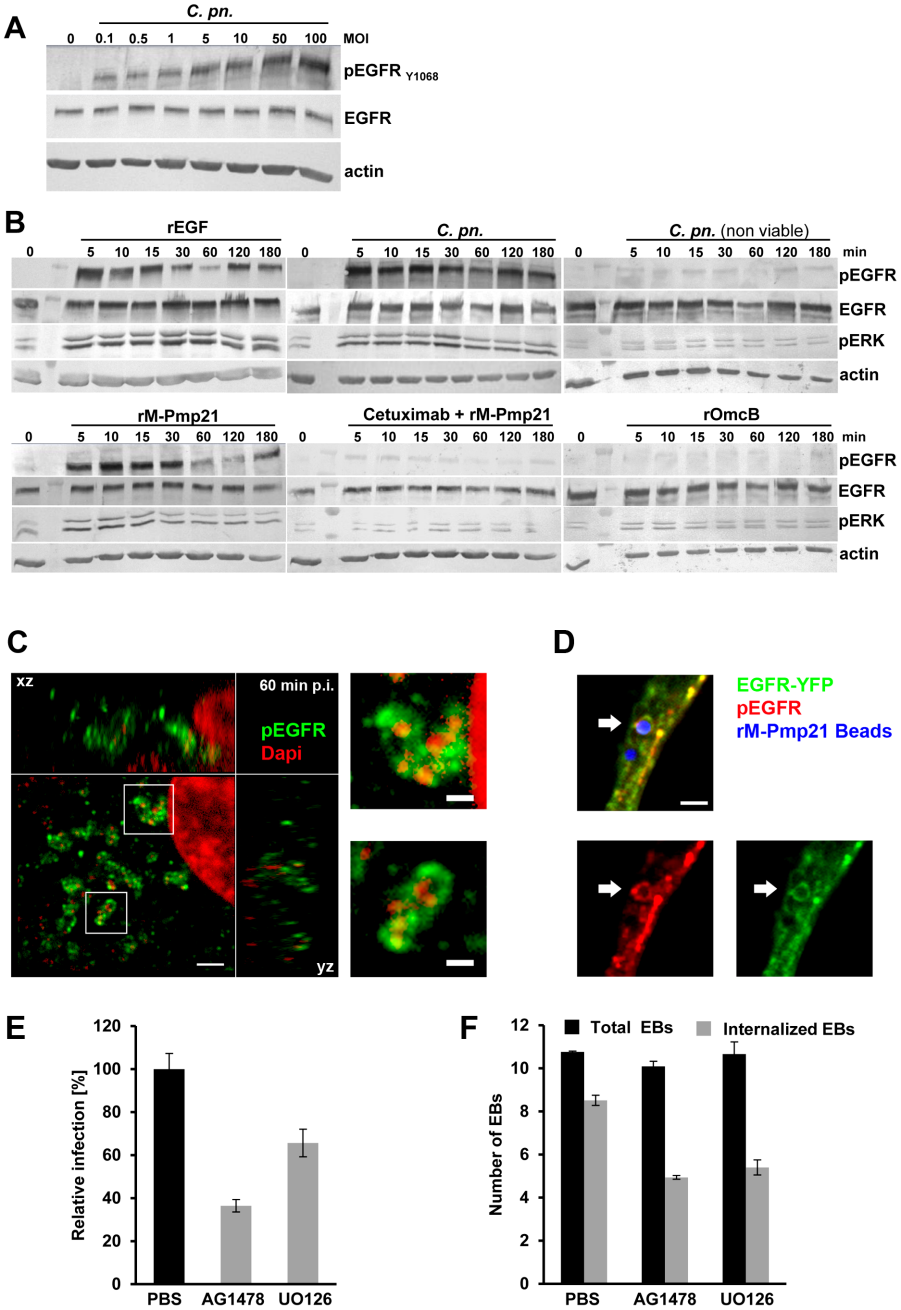
EGFR signaling is activated by *C. pneumoniae* EBs and recombinant Pmp21. (**A**) Kinetics of *C. pneumoniae* EB-induced phosphorylation of EGFR. HEp-2 cells were left uninfected (0) or infected with increasing numbers of *C. pneumoniae* EBs (MOI) for 60 min. The immunoblots show total levels of EGFR (EGFR) and levels of activated receptor (pEGFR, phosphorylated at Tyr1068) detected as described in Experimental Procedures. (**B**) Time courses of EGFR activation by rEGF (100 ng/ml), purified (viable and non-viable) *C. pn.* EBs (MOI 5), rM-Pmp21 (100 µg/ml) in the presence or absence of Cetuximab (5 µg/ml), and rOmcB (100 µg/ml). HEp-2 cells were serum-starved for 12 h at 37°C, shifted to 4°C for 10 min before the addition of bacteria or recombinant proteins, then incubated further at 37°C. The immunoblots show overall levels of EGFR (EGFR), activated EGFR (pEGFR) and activated ERK (pERK), the downstream MAP kinase. Actin served as loading control. (**C–D**) Confocal spinning-disk microscopy of *C. pneumoniae* EBs (**C**) or rM-Pmp21-coated latex beads (**D**) surrounded by ring-like structures of activated EGFR (pEGFR_Y1068_). Scale bars 1 µm. (**C**) HEp-2 cells were infected with *C. pneumoniae* for 60 min. Two examples of internalized EBs surrounded by EGFR (white boxes) are shown at a higher magnification on the right. (**D**) Beads coated with rM-Pmp21 were added to EGFR-YFP-expressing CHO-K1 cells and incubated for 4 h at 37°C. The arrow marks a bead in the focal plane harboring EGFR and pEGFR rings. (**E**) EGFR inhibitors reduce infection by *C. pneumoniae* EBs. Confluent HEp-2 cells were pretreated for 2 h with PBS, AG1478 or UO126, and infected with *C. pneumoniae* EBs (MOI 1) for 48 h. The data represent the means of four independent experiments. (**F**) Internalization of *C. pneumoniae* EBs (MOI 1) by HEp-2 cells treated for 2 h with PBS, AG1478 or UO126. Internalized EBs were quantified as described in Experimental Procedures. The data represent the means of five independent experiments.

Serum-starved HEp-2 cells were infected at increasing MOIs with *C. pneumoniae* EBs for 60 min. Progressive, dose-dependent autophosphorylation of Y1068 was detected, while total amounts of EGFR remained unchanged (Fig. 5A). Next we determined the kinetics of EGFR activation by incubating HEp-2 cells with recombinant EGF, viable or non-viable *C. pneumoniae* EBs, rPmp21 or rOmcB protein (Fig. 5B). The natural ligand EGF induced rapid phosphorylation of Y1068. Viable EBs also evoked strong autophosphorylation of Y1068, beginning after 5 min of incubation and lasting for 3 h. HEp-2 cells incubated with heat-inactivated EBs showed no phosphorylation. Most strikingly, incubation of HEp-2 cells with rM-Pmp21 also triggered fast phosphorylation of Y1068. As expected, rM-Pmp21-induced EGFR activation was abrogated by co-incubation with the receptor-blocking antibody cetuximab. Importantly, exposure of human cells to a different chlamydial adhesin, rOmcB, did not activate the receptor (Fig. 5B). These data indicate that binding of soluble Pmp21 also activates EGFR.

Next we tested whether catalytically activated EGFR was recruited to endocytic vesicles containing *C. pneumoniae* EBs 60 min pi using immunofluorescence microscopy. Endogenous EGFR phosphorylated at Y1068 was found to colocalize in ring-like structures at bacterial entry sites (Fig. 5C). Quantification revealed that 85% of EB signals colocalized with activated EGFR signals, while this was only the case for 20% of the human transferrin receptor hTfR (Fig. S3A+B). This confirms that binding of *C. pneumoniae* activates EGFR and that the activated receptor specifically clusters with the bacteria during internalization. To test for Pmp21-induced EGFR activation directly, we followed the fate of rM-Pmp21-coated beads upon incubation with CHO-K1 cells transfected with EGFR-YFP. Internalized Pmp21 beads were surrounded by ring-like structures bearing EGFR-YFP phosphorylated at Y1068 EGFR, proving that Pmp21 both binds and activates EGFR (Fig. 5D).

That activation of EGFR is needed for EB uptake was demonstrated with the EGFR-specific kinase inhibitor AG1478. Pretreatment of host cells for 2 h with AG1478 (Fig. 5E) reduced infectivity by 63%, which correlates well with the 41% reduction in EB internalization, while EB attachment was unaffected (Fig. 5F). Hence EGFR kinase activity is indeed important for endocytosis of chlamydial EBs.

Signaling by EGFR activates the MAP kinase pathway, which results in phosphorylation of ERK1/2 (Fig. 5B; rEGF) [17]. Like rEGF, *C. pneumoniae* EBs triggered rapid activation of ERK1/2, which peaked at 30 min, in agreement with previous findings [6]. Furthermore, rM-Pmp21 induced ERK1/2 phosphorylation as well, while incubation with rM-Pmp21 and cetuximab, or with rOmcB, failed to activate the kinases (Fig. 5B). These results strongly suggest that Pmp21 on the EB surface binds to EGFR, triggering its activation and inducing downstream signaling just as EGF does.

To test whether the MAP kinase pathway facilitates invasion by *C. pneumoniae*, we blocked the MEK1/2 kinase, which phosphorylates ERK, by pre-incubating HEp-2 cells with the inhibitor UO126. Indeed, inhibition of MEK1/2 activity reduced subsequent chlamydial infectivity by 33% (Fig. 5E). This was entirely due to diminished internalization of chlamydial particles (down to 63%), as binding of the bacteria to the host cell was not affected (Fig. 5F) [6]. Thus, both kinase inhibitors affect the internalization of EBs, implying that uptake of EBs is critically dependent on downstream signaling cascades.

### Expression of a functional EGFR in a receptor-negative cell line increases susceptibility to infection by *C. pneumoniae*

**Figure 6:**
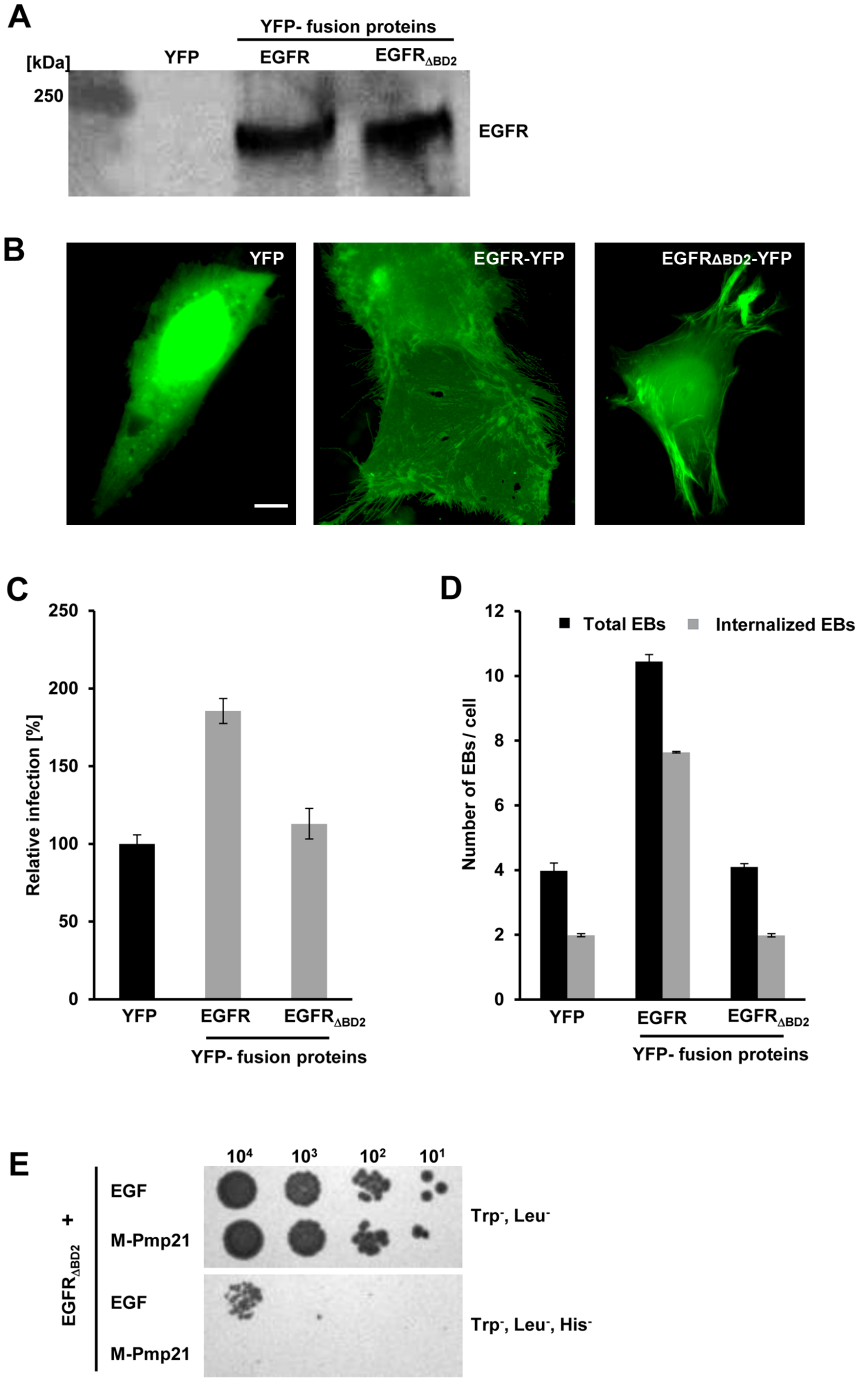
A functional EGF-binding domain in EGFR is essential for infection by *C. pneumoniae*. (**A–D**) EGFR-deficient CHO-K1 cells were transfected with EGFR-YFP, EGFR_ΔBD2_-YFP or YFP alone for 24 h. (**A**) EGFR expression was quantified by immunoblot analysis of lysates of transfected cells using an anti-EGFR antibody. (**B**) Subcellular localization of YFP and the two EGFR-YFP constructs by direct immunofluorescence of transfected CHO-K1 cells. Bar 5 µm. (**C**) Susceptibility of transfected CHO-K1 cells to infection with *C. pneumoniae* GiD. Cells were incubated with EBs (MOI 1) for 48 h. Inclusions were quantified using an antibody directed against the inclusion membrane protein Cpn0147. The data represent the means of four independent experiments. (**D**) Internalization of *C. pneumoniae* EBs (MOI 1) by CHO-K1 cells transfected with YFP, EGFR-YFP or EGFR_ΔBD2_-YFP. Numbers of internalized EBs were determined in positively transfected cells only. The data represent the means of five independent experiments. (**E**) Y2H analysis of EGFR_ΔBD2_/EGF and EGFR_ΔBD2_/M-Pmp21 interactions. Serial dilution patch test of 10^1^–10^4^ cells on selective medium.

To further define the role of EGFR in *C. pneumoniae* infection, we ectopically expressed an EGFR-YFP fusion in EGFR-negative CHO-K1 cells (Fig. 6A) [15]. CHO cells are capable of expressing EGFR and activating downstream signaling cascades, as ERK phosphorylation has been shown in these cells [23]. YFP expressed on its own was detectable in the cytosol and accumulated in the nucleus (Fig. 6B, YFP). In contrast, EGFR-YFP localized to the plasma membrane (including filopodia) and none was found in the nucleus (Fig. 6B, middle panel; Fig. S5A). Expression of EGFR-YFP in CHO-K1 cells increased their susceptibility to *C. pneumoniae* by 180% relative to YFP-expressing cells (Fig. 6C). The increase in infection was associated with a 265% increase in adhesion and a 365% rise in internalization (Fig. 6D). Hence the presence of human EGFR makes hamster cells more sensitive to invasion by *C. pneumoniae*. Furthermore, while preincubation of EGFR-expressing CHO-K1 cells with the EGFR kinase inhibitor AG1478 did not alter the number of EBs attached to the cells, it reduced the number of internalized EBs by 30% (Fig. S5C). As anticipated, blocking the EGFR ligand binding site in EGFR-positive CHO cells with cetuximab reduced the number of associated EBs by 25% and internalized EBs by 55% (Fig. S5C).

### Pmp21 binding to EGFR requires the L2 domain of the receptor

Since the anti-EGFR antibody cetuximab, which blocks the EGF-binding site, also blocks EB binding (see Fig. 4), EGF and Pmp21 might recognize overlapping binding sites. The EGF-binding pocket of EGFR is formed by four subdomains, two L (ligand-binding) and two CR (cysteine-rich) regions [24], [16]. To analyze the role of this domain in infection by *C. pneumoniae*, CHO cells were transfected with a truncated version of EGFR (EGFR_ΔBD2_) that lacks L2. EGFR_ΔBD2_ and wild-type EGFR were expressed at comparable levels (Fig. 6A), and EGFR_ΔBD2_ was detected in the cytoplasm, the nucleus and on the plasma membrane (Fig. 6B, right panel; Fig. S5A). Importantly, similar amounts of both wild-type EGFR and EGFR_ΔBD2_ were detected on the surface of transfected CHO cells (Fig. S5A, B). In CHO cells expressing EGFR_ΔBD2_, levels of adhesion and internalization of *C. pneumoniae* EBs were almost identical to those in YFP-expressing controls (Fig. 6C, D). Moreover, preincubation of these CHO cells with either AG1478 or cetuximab did not affect either EB association or EB internalization levels (Fig. S5C). These data prove that a functional EGFR is needed for successful infection by *C. pneumoniae*.

Finally we asked whether domain L2 of EGFR is essential for its interaction with Pmp21 in Y2H experiments. Deletion of L2 markedly weakened EGFR's interaction with its ligand EGF, in agreement with published data [11], However, M-Pmp21 was now completely unable to support growth on selective media when co-expressed with EGFR_ΔBD2_ (Fig. 6E). Thus, Pmp21 also binds EGFR, at least in part, via the L2 domain.

### Interaction of EGFR with adaptor proteins Grb2 and c-Cbl is essential for infection by *C. pneumoniae*

**Figure 7:**
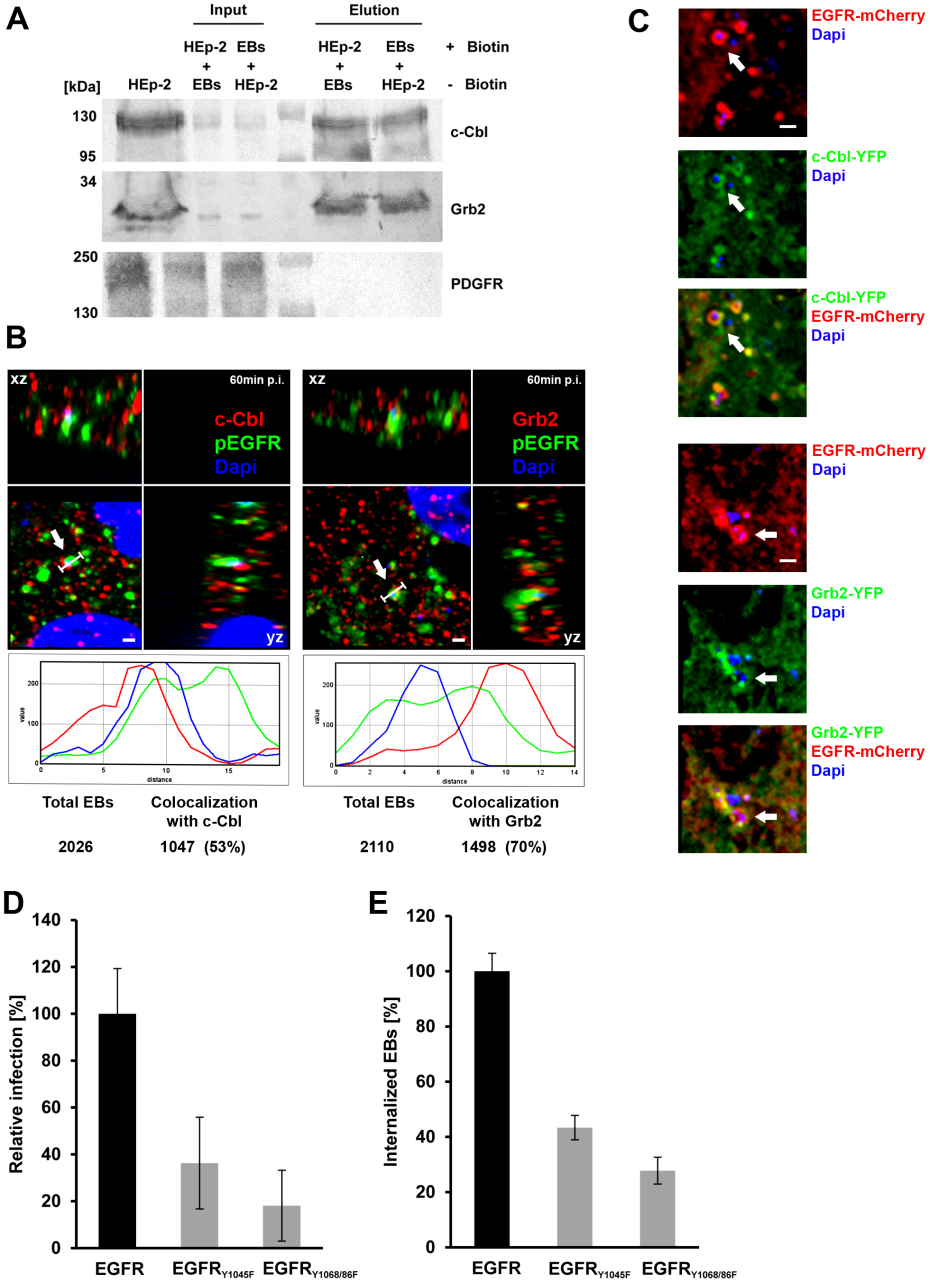
The EGFR adaptor proteins c-Cbl and Grb2 colocalize with invading *C. pneumoniae* EBs. (**A**) c-Cbl and Grb2 specifically interact with invading *C. pneumoniae* EBs. Adaptor proteins c-Cbl and Grb2 were affinity-enriched from surface-biotinylated HEp-2 cells incubated with non-biotinylated *C. pneumoniae* EBs, or *vice versa*, for 60 min at 37°C. The eluted proteins were subjected to immunoblot analysis with specific antibodies against c-Cbl, Grb2 or PDGFRβ. Equal amounts of input and elution samples were loaded onto the SDS/PAGE. (**B–C**) c-Cbl and Grb2 colocalize with EGFR and invading EBs. (**B**) Confocal spinning-disk microscopy of *C. pneumoniae* EBs infecting HEp-2 cells for 60 min at 37°C. The arrow marks a single DAPI-stained EB (blue) colocalizing with antibody-stained endogenous EGFR (green) and endogenous c-Cbl (red) or endogenous Grb2 (red). Bar 1 µm. Below each picture an intensity scan along the indicated white line is shown (blue – DAPI signal; green – EGFR signal; red – c-Cbl or Grb2 signal). Quantification of colocalization of EBs stained by DAPI and c-Cbl or Grb2 signals is shown below. The data represent the means of five independent experiments. (**C**) Colocalization of transfected EGFR-mCherry with either c-Cbl-YFP or Grb2-YFP and invading *C. pneumoniae* EBs in CHO-K1 cells. The arrow marks a single EB surrounded by an EGFR-mCherry signal and either c-Cbl-YFP or Grb2-YFP signals. Bar 1 µm. (**D**) Functional interaction between EGFR and c-Cbl and Grb2 is essential for infection by *C. pneumoniae*. CHO-K1 cells were transfected with wt EGFR or the mutant EGFR_Y1045F_ (docking site for c-Cbl) or EGFR_Y1068/1086F_ (docking site for Grb2) and 24 h later infected with *C. pneumoniae* for 48 h. Inclusions were quantified using an antibody against Cpn0147 (n = 4). (**E**) Internalization of *C. pneumoniae* EBs (MOI 1) by CHO-K1 cells transfected with EGFR-YFP or EGFR_Y1045F_ or EGFR_Y1068/1086F_. Numbers of internalized EBs were determined in positively transfected cells only. The data represent the means of five independent experiments.

EGFR activation leads to recruitment of the adaptor protein Grb2 and the ubiquitin ligase c-Cbl [25]. Grb2 binds activated EGFR at phosphotyrosines 1068/1086, and induces ERK1/2 signaling via Ras and Raf, rather than the MAP kinase pathway. It also recruits c-Cbl, which is involved in receptor endocytosis. We tested whether EB binding to EGFR also results in recruitment of Grb2 and c-Cbl, using an analogous affinity approach to that used to detect the interaction of Pmp21 with EGFR (Fig. 2C). Grb2 and c-Cbl were both significantly enriched in affinity eluates compared to the input controls. PDGFRβ which is implicated in the *C. trachomatis* infection was absent from the eluate (Fig. 7A). The recruitment of Grb2 and c-Cbl by EBs was corroborated by microscopy. HEp-2 cells incubated with bacteria for 60 min revealed specific colocalization of bacterial DNA with endogenous EGFR and endogenous c-Cbl or Grb2 in 53% and 70% respectively, as shown in Fig. 7B. Recruitment of Grb2 and c-Cbl to the invading EB was also documented in CHO-K1 cells transfected with EGFR-mCherry and either c-Cbl-YFP or Grb2-YFP. EGFR and Grb-2 or c-Cbl formed ring- or patch-like structures surrounding or otherwise associated with the bacteria (Fig. 7C).

We also asked whether blockage of the receptor-adaptor protein interaction would negatively affect chlamydial infection. Transfection of a point-mutated EGFR construct (Y1068F, Y1086F) known to interfere with Grb2 binding into CHO-K1 cells reduced EB internalization by 72% and the subsequent *C. pneumoniae* infection by 82%. Similarly, the Y1045F mutation in EGFR, which is known to affect the interaction of EGFR with c-Cbl, resulted in reduction of internalized EBs by 56% and in a reduction in infection by 64% (Fig. 7D+E). These results underline the importance of EGFR and its adaptor proteins for successful infection by *C. pneumoniae*.

## Discussion

*Chlamydiae* are obligate intracellular pathogens, and invasion of eukaryotic host cells is essential for their survival. Generally, initial association with target cells occurs via the chlamydial adhesin OmcB, which interacts with heparan sulfate glycosaminoglycans [26]. Three members of the large, heterogeneous Pmp family have recently been characterized as adhesins that mediate attachment of *C. pneumoniae* to epithelial cells, and are important for subsequent infection [8]. Here we show that *C. pneumoniae* Pmp21 also acts as an invasin (Fig. 1). We show that Pmp21 binds to EGFR (Fig. 2) and activates EGFR (Fig. 5) and that the interaction is required for internalization of infectious EBs (Figs. 3, 4). Interestingly Pmp21 is the first pathogen-derived EGFR ligand shown to interact directly with EGFR. While EGFR activation has been associated with exposure to a number of bacterial and viral pathogens including influenza and HCMV viruses, a direct role for EGFR as a pathogen receptor has remained controversial until now [27], [28], [29].

In our pull-down experiments using biotinylated Pmp21, we identified EGFR in three electrophoretically distinguishable forms (all larger than 170 kDa) (Fig. 2A). The fact that only peptides from EGFR but not from the other three members of the ErbB family of receptors were identified by MS suggests that Pmp21 interacts with EGFR homodimers. Different biochemical and cell biological approaches have been used here to show the specificity of the EGFR-Pmp21 interaction. Binding of Pmp21 to EGFR was also verified by Y2H assays (Fig. 2E), which show an interaction level similar to that found for EGFR with its natural ligand EGF [11]. The Y2H data also strongly argue that the interaction of Pmp21 with EGFR is direct and is not mediated by one of the receptor's natural ligands. The complete loss-of-function phenotype of the EGFR_ΔBD2_, which lacks the second EGF ligand-binding domain L2, further indicates that EGF and Pmp21 may well use (at least partially) overlapping binding pockets. EGFR recognizes a specific motif of three disulfide bonds formed by six conserved Cys residues [30], but there is no indication that Pmp21 can form such a typical EGF-like fold. The rate of adhesion and also of internalization of Pmp21-beads seems to be significantly lower than that found for invasin-beads (Fig. 1A+B) suggesting a lower affinity of the M-Pmp21 ligand to the EGF receptor. One may speculate that the Pmp21-EGFR interaction stabilizes the EB-host cell contact and is thus likely to be relevant for the activation of the bacterial Type III system required for secretion of early effector proteins like Tarp.

The Pmp family in *C. pneumoniae* has 21 members, and it is intriguing to speculate that Pmp6 and Pmp20 [8], and possibly other family members, might also act as invasins by binding and activating EGFR. Pmp proteins show little overall similarity, but all have multiple repeats of the tetrapeptide motifs GGA(I,L,V) and FxxN, and these might be relevant for the recognition and/or activation of EGFR. Thus *Chlamydiae* may optimize their chances of reaching their intracellular niche by using multiple adhesins and a ubiquitously expressed cellular receptor.

Importantly, our findings suggest a direct dependence between the levels of EGFR on the cell surface and susceptibility to infection by *C. pneumoniae*. Depletion of the receptor in HEp-2 cells by specific siRNA, addition of EGF or blocking of the ligand-binding pocket with an anti-EGFR antibody significantly reduced both EB attachment and infection (Fig. 4). Conversely, expression of EGFR in normally receptor-deficient cells increased EB attachment and internalization, and susceptibility to infection (Fig. 3D). However, the requirement for EGFR in *C. pneumoniae* entry is not absolute, which suggests that additional unidentified uptake mechanisms operate. Interestingly, depletion of EGFR in HeLa cells did not reduce infection by another chlamydial species, pointing to differences in receptor usage between chlamydial species [31]. This receptor specificity is supported by data showing that rPmp21 is unable to reduce a *C. trachomatis* infection (Becker and Hegemann, unpubl.). Here we show that the PDGF receptor previously implicated in the attachment und uptake of a different chlamydial species shows no interaction with Pmp21 (Figs. 2C, 2D). Thus although the Pmp proteins from different chlamydial species share certain sequence similarities they seem to use different receptors. Several other human cell surface proteins (apolipoprotein E4, mannose/mannose-6-phosphate receptor, PDI/estrogen receptor, FGFR) have been implicated in adhesion of certain *Chlamydia* spp, but a direct interaction between any of these receptors and chlamydial EBs has yet to be shown [26], [32].

Binding of *C. pneumoniae* EBs or recombinant Pmp21 to HEp-2 or transfected CHO cells results in rapid EGFR activation, which colocalized with attached and internalized EBs and clustered around Pmp21-coated beads (Figs. 3, 5C+D). EGFR activation is necessary for chlamydial EB entry, as incubation with the EGFR kinase inhibitor AG1478 reduced the number of internalized EBs. EGFR phosphorylation at Y1068 and Y1086 induces recruitment of the adapter protein Grb2, which then allows binding of c-Cbl to EGFR Y1045. This protein complex enables EGFR internalization via clathrin-dependent as well as -independent endocytosis [21], [33]. Remarkably our biochemical and microscopical data show that both Grb2 and c-Cbl co-localized with wild-type EGFR and internalized Chlamydia (Fig. 7A+B), and this interaction was critical for infection, as infectivity was reduced 5-fold when an Y1068/1086 EGFR mutant was expressed (Fig. 7D), and this reduction is almost identical to the 5-fold reduction in rates of internalization previously measured for this EGFR mutant form [34]. Interestingly, the invading *C. pneumoniae* EBs did not colocalize with the transferrin receptor, a classical marker for the clathrin-derived endocytic system (Fig. S3A+B), which is compatible with new data suggesting that entry of *C. pneumoniae* may not depend on clathrin but on lipid rafts, although the molecular details remain to be clarified [35], [36].

Our data show that *C. pneumoniae* recruits the EGFR/Grb2/c-Cbl complex via Pmp21 and activates the ERK1/2 kinases, and thus confirm and extend data indicating that *C. pneumoniae* infection activates SHC, MEK1/2, ERK and PI3K [6]. Activated PI3K can modulate actin dynamics [37]. Blocking MEK1/2 or PI3 kinase activity reduced EB internalization and infection but not EB binding, proving the relevance of this EGFR-mediated signaling pathway for chlamydial entry [6] (this work). Finally the *C. pneumoniae* infection leads to FAK1 activation [6], and it is conceivable that EGFR activation by Pmp21 induces phosphorylation of FAK, which also is involved in cytoskeleton regulation. Thus binding of Pmp21-coated beads or infectious EBs (via Pmp21) to EGFR induces receptor activation, and subsequent endocytosis of the bacterial cell. The latter process probably requires not only downstream signaling cascades in the host cell, but is also modulated by the secretion of bacterial effector proteins like Tarp, which contributes to actin cytoskeleton reorganization at the EB entry site [38]. The accumulation of active EGFR around the inclusion containing endocytosed EBs 60 min post infection (see Fig. 5C), points to a role for (active) EGFR beyond the entry process *per se*.

It should be emphasized that our results do not exclude the involvement of other yet unidentified chlamydial adhesins/invasins and host cell proteins in the entry process. A complete understanding of the molecular interplay between pathogen and host is a prerequisite for the development of novel efficient strategies to prevent chlamydial diseases.

## Materials and Methods

### Inhibitors, antibodies and reagents

The EGFR kinase inhibitor AG1478, the MEK1/2 inhibitor U0126 and monoclonal antibodies directed against phosphorylated EGFR (Y1068, rabbit) and phosphorylated ERK (p44/42, mouse) were obtained from Cell Signaling. The neutralizing antibody cetuximab (Merck) was kindly provided by Dr. B. Homey. Polyclonal antibodies against EGFR, c-Cbl, Grb2, PDGFRβ and Integrin-β1 (CD29) were purchased from Santa Cruz, the β-actin antibody and recombinant EGF from Sigma, the hTfR antibody and Wheat Germ Agglutinin-Alexa594 (WGA) from Invitrogen and DTSSP from Thermo Scientific. The anti-GFP antibody was purchased from GeneTex. The anti-invasin antibody was donated by Petra Dersch. The antibody against recombinant M-Pmp21 and Momp have been described elsewhere [8], [39]. All siRNA's were obtained from Santa Cruz Biotechnology, EGFR (sc-29301), and Control NT-siRNA-A (sc37007).

### Bacterial strains and cell lines

*C. pneumoniae* GiD was propagated in the cell lines HEp-2 (ATCC: CCL-23), HeLa229 (ATCC: CCL-2.1) and CHO-K1 (ATCC: CCL-61) [40], [41], [42], [43]. HEp-2 and HeLa229 cells were cultured in DMEM medium, and CHO-K1 cells in Ham's F12-K nutrient mixture medium, each supplemented with 10% fetal calf serum (FCS; Invitrogen). Chlamydial elementary bodies (EBs) were purified using a 30% gastrographin solution (Schering) and, where appropriate, incubated for 10 min at 100°C and chilled on ice to inactivate infectivity. *Escherichia coli* strain XL-1 Blue (Stratagene) was used for protein expression and plasmid amplification, and *Saccharomyces cerevisiae* for two-hybrid experiments. Cloning in yeast was carried out by *in vivo* homologous recombination.

### Biotin pull-down experiments

Recombinant proteins or purified chlamydial particles were biotinylated with NHS-SS-biotin and incubated with non-biotinylated human epithelial cells. Interacting proteins were crosslinked and isolated by passage over a NeutrAvidin column. For further details see Supplemental Experimental Procedures.

### Co-immunoprecipitation from infected cells

EGFR/M-Pmp21 or PDGFR were immunoprecipitated from HEp-2 cells infected with *C. pneumoniae* EBs at 60 min post-infection using specific antibodies. For further details see Supplemental Experimental procedures.

### Alteration of EGFR expression by transfection procedures

Expression of YFP or mCherry-tagged EGFR variants was carried out in EGFR-deficient CHO-K1 cells, while depletion of EGFR by specific siRNA was performed by transfection of HeLa229 cells. Expression levels of EGFR were monitored by immunoblotting or fluorescence microscopy. For further details see Supplemental Experimental procedures.

### Internalization of chlamydial particles

Internalization of *C. pneumoniae* EBs into epithelial cell lines expressing endogenous EGFR or YFP-tagged EGFR variants was analyzed in the presence of various EGFR inhibitors, and the ratio of external to internalized bacteria was quantified by fluorescence microscopy. For further details see Supplemental Experimental procedures.

### Binding and internalization of protein-coated latex beads

Adhesion assays with fluorescent protein-coated latex beads were performed with a five-fold excess of beads over cells as described previously [8]. Beads attached to epithelial cells were either counted by optical microscopy, or analyzed by flow cytometry in a FACSAria (BD Biosciences) after washing (with PBS) and dissociation (with Cell Dissociation solution; Sigma). For internalization studies adhesion assays were carried out for 1 h at 4°C or 4 h at 37°C. Cells were washed twice with PBS and fixed with 3% formaldehyde for 20 min. External beads were stained (without permeabilization) with primary antibodies directed against the protein coupled to them. Internalization efficiency was determined by subtracting the numbers of external beads from the total numbers found associated with samples of 10^3^ cells.

## Supporting Information

Figure S1 **Internalization of M-Pmp21 or invasin-coated latex beads by HEp-2 cells (Related to** **Figure 1B** **).** Confocal spinning-disc MIP (Maximum Intensity Projection) images of HEp-2 cells that had been incubated with recombinant GST-, invasin-, GroEL- or M-Pmp21-coated green fluorescent latex beads (5 beads/cell) for 1 h at 4°C or 4 h at 37°C. All external beads were stained in red using specific antibodies directed against the protein coupled to the beads. Internalized beads are not accessible to the antibodies and emit green fluorescence. Arrows mark internalized beads at 37°C. Bar 1 µm.
(TIF)

Figure S2 **Identification of EGFR as interaction partner for rM-Pmp21 (Related to** **Figure 2A** **).** (**A**) Schematic depiction of labeling protocol for surface proteins that interact with recombinant M-Pm21 protein. (**B**) Binding proteins were eluted from NeutrAvidin columns, and fractionated by SDS-PAGE. Bands were then excised from the gel and trypsinized, and the resulting peptides were identified by mass spectroscopy. Band numbers match numbers shown in Figure 2A. A protein was designated as a significant hit if the peptide fingerprint matched that predicted for the listed protein with a probability of p<0.05. In Band 5 insignificant contamination with L1 CAM (asterisk) was identified. In Band 6 significant contamination with FLNA was observed.
(TIF)

Figure S3 **The human transferrin receptor does not colocalize with internalized bacteria (Related to** **Figures 2C** **and** **5C** **).** (**A**) Confocal spinning-disk images of HEp-2 cells infected with *C. pneumoniae* EBs (MOI 1) for 60 min. Internalized bacteria stained with DAPI (red) are surrounded by activated EGFR, stained with a phospho-EGFR antibody (green). Human transferrin receptor (stained in blue) does not colocalize with the internalized bacteria, as shown in the fluorescence intensity plot (panel below image) generated from a section through the marked area (white arrow). Bar: 1 µm. (**B**) Quantification of colocalization of EBs with activated EGFR (pEGFR) or human transferrin receptor (hTfR) at 60 min pi. EBs were stained by DAPI, pEGFR and hTfR with specific antibodies. The data represent the means of five independent experiments.
(TIF)

Figure S4 **Interaction of EGFR and M-Pmp21 confirmed by yeast two-hybrid analysis (Related to** **Figure 2E** **).** (**A**) Serial dilution patch tests of yeast two-hybrid clones. 10^4^ - 10^1^ yeast cells were patched on selective (Trp^−^, Leu^−^) and on low-stringency medium (Trp^−^, Leu^−^, His^−^). The integrin-β1 construct showed autoactivation (*). (**B**) Immunoblot analysis of yeast cells expressing EGFR or EGFR_ΔBD2_ detected with an EGFR antibody (left). Expression of EGF and M-Pmp21 yeast two-hybrid constructs was detected with a penta-His antibody (right).
(TIF)

Movie S1 **3D model of EGFR-YFP surrounding internalized** ***C. pneumoniae*** **EBs (Related to** **Figure 3D** **).** Images taken from the boxed area of Figure 3D were imported into ImageJ software to calculate a 3D model of all focal planes based on a maximal intensity projection. The movie was generated by 360° rotation of the model.
(AVI)

Figure S5 **Inhibition of EGFR function in EGFR-expressing CHO-K1 cells reduces rates of EB internalization (Related to** **Figure 6D** **).** (**A**) Confocal spinning-disk images of CHO-K1 cells transfected with EGFR-YFP or EGFR_ΔBD2_-YFP fixed with formaldehyde. Cells were either permeabilized (+ Saponin) or left untreated (− Saponin) and stained with an anti-GFP antibody (red) to detect the C-terminal GFP-tag. Bar: 10 µm. (**B**) Quantification of the amounts of EGFR-YFP and EGFR_ΔBD2_-YFP expressed on the surface of transfected CHO-K1 cells. Fixed cells were stained first with a mouse antibody that recognizes the EGFR ectodomain (aa 6–273) followed by incubation with FITC-conjugated anti-mouse antibody. Samples of 10,000 transfected CHO-K1 cells each were quantified for cell surface fluorescence by FACS analysis. (**C**) CHO-K1 cells transfected with EGFR constructs as described in Figure 6A–D were pretreated with the EGFR kinase inhibitor AG1478 or the blocking antibody cetuximab before being exposed to *C. pneumoniae* EBs The total number of EBs (total) associated with cells and the number of internalized EBs (internalized EBs) were determined as described previously. The data represent the means of four independent experiments.
(TIF)

Text S1 **Supporting information includes Supplemental Experimental Procedures and a list of relevant Gene Accession Numbers.**
(DOCX)
