# Supplementary figures and images for: The Chlamydia pneumoniae Invasin Protein Pmp21 Recruits the EGF Receptor for Host Cell Entry
Source: PLoS Pathog. 2013 Apr 25;9(4):e1003325. doi: 10.1371/journal.ppat.1003325 (PMC3635982; doi:10.1371/journal.ppat.1003325)

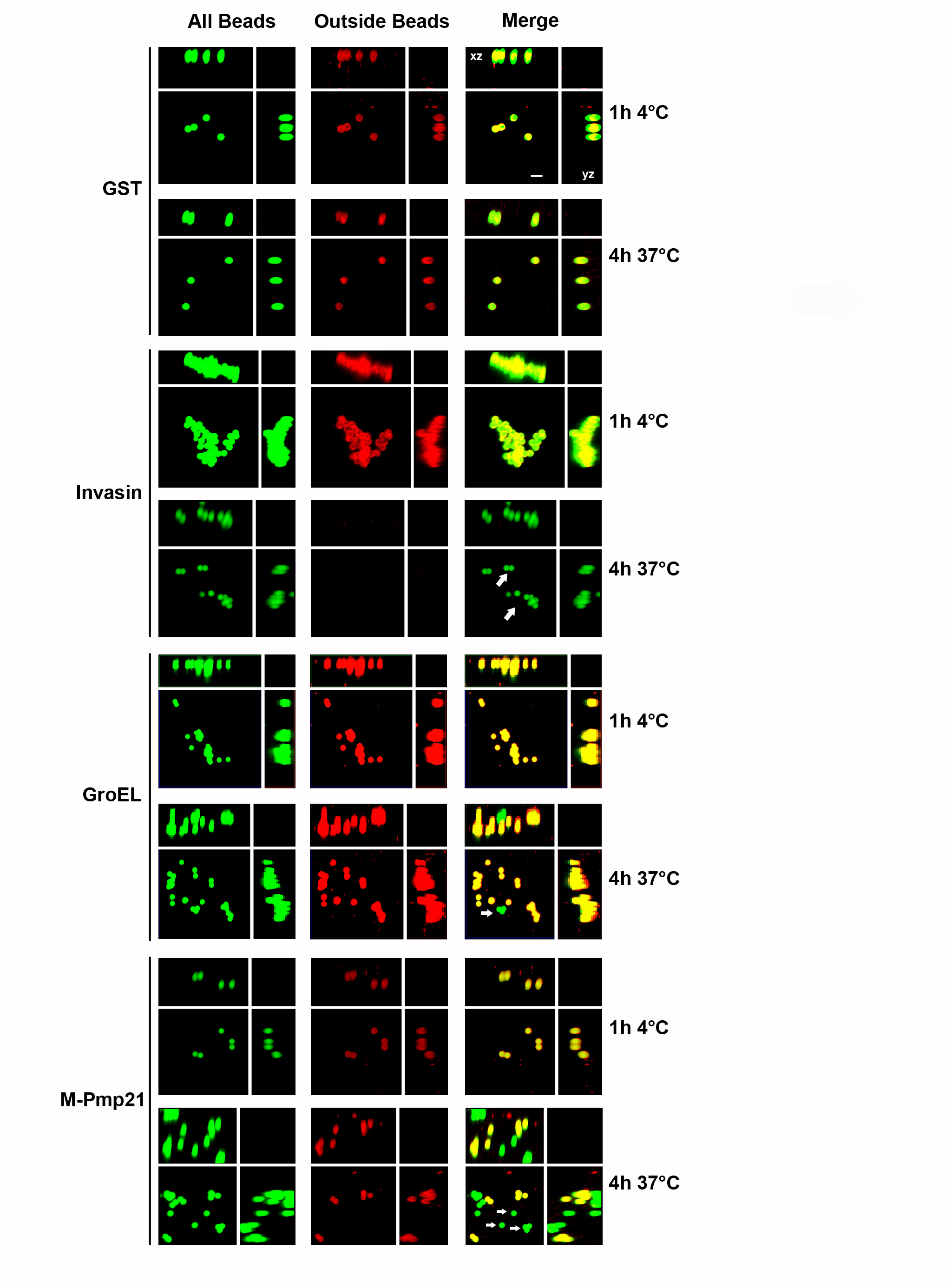

Supplement: Figure S1 — Internalization of M-Pmp21 or invasin-coated latex beads by HEp-2 cells (Related to Figure 1B ). Confocal spinning-disc MIP (Maximum Intensity Projection) images of HEp-2 cells that had been incubated with recombinant GST-, invasin-, GroEL- or M-Pmp21-coated green fluorescent latex beads (5 beads/cell) for 1 h at 4°C or 4 h at 37°C. All external beads were stained in red using specific antibodies directed against the protein coupled to the beads. Internalized beads are not accessible to the antibodies and emit green fluorescence. Arrows mark internalized beads at 37°C. Bar 1 µm. (TIF) [file ppat.1003325.s001.tif]

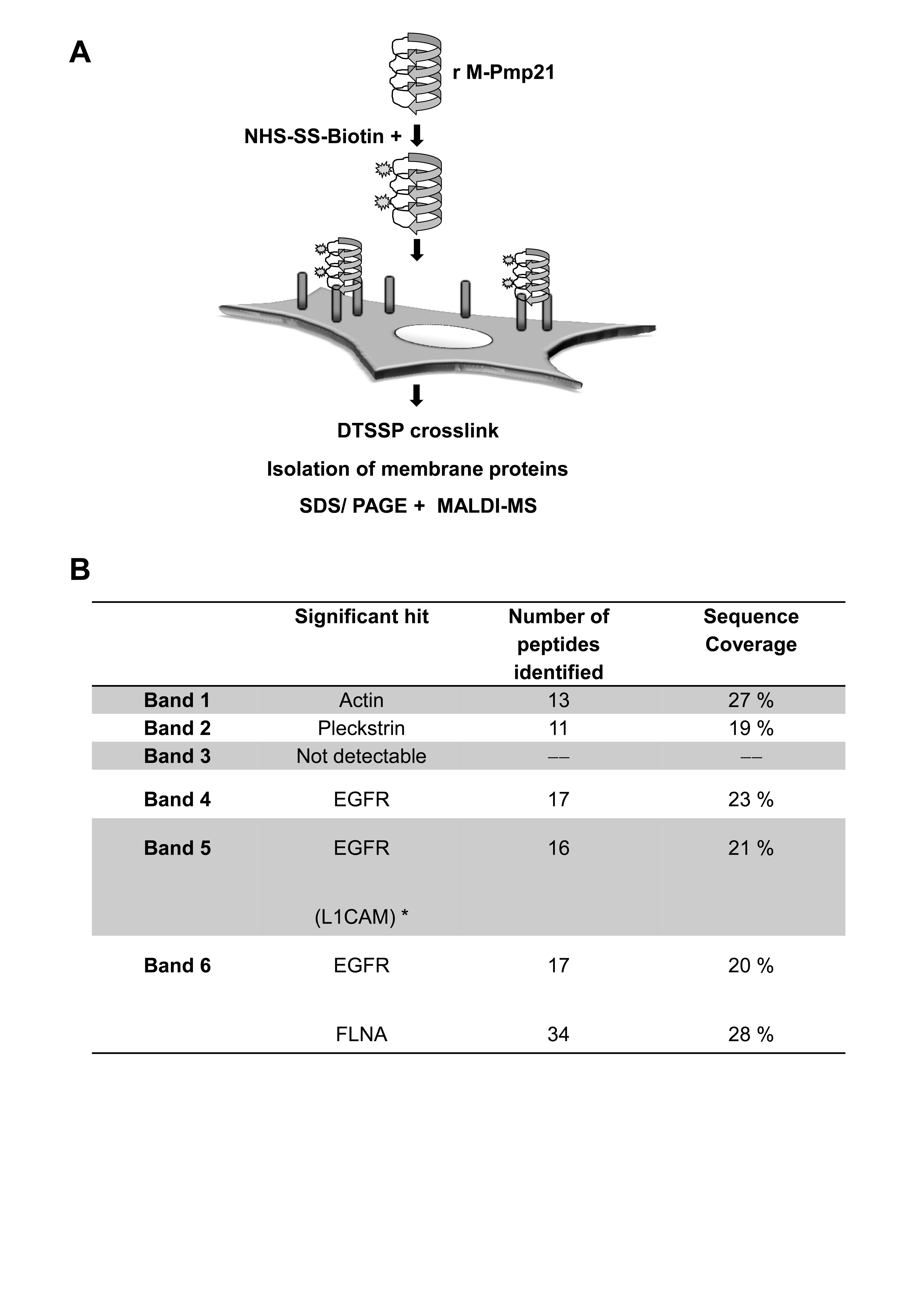

Supplement: Figure S2 — Identification of EGFR as interaction partner for rM-Pmp21 (Related to Figure 2A ). (A) Schematic depiction of labeling protocol for surface proteins that interact with recombinant M-Pm21 protein. (B) Binding proteins were eluted from NeutrAvidin columns, and fractionated by SDS-PAGE. Bands were then excised from the gel and trypsinized, and the resulting peptides were identified by mass spectroscopy. Band numbers match numbers shown in Figure 2A. A protein was designated as a significant hit if the peptide fingerprint matched that predicted for the listed protein with a probability of p<0.05. In Band 5 insignificant contamination with L1 CAM (asterisk) was identified. In Band 6 significant contamination with FLNA was observed. (TIF) [file ppat.1003325.s002.tif]

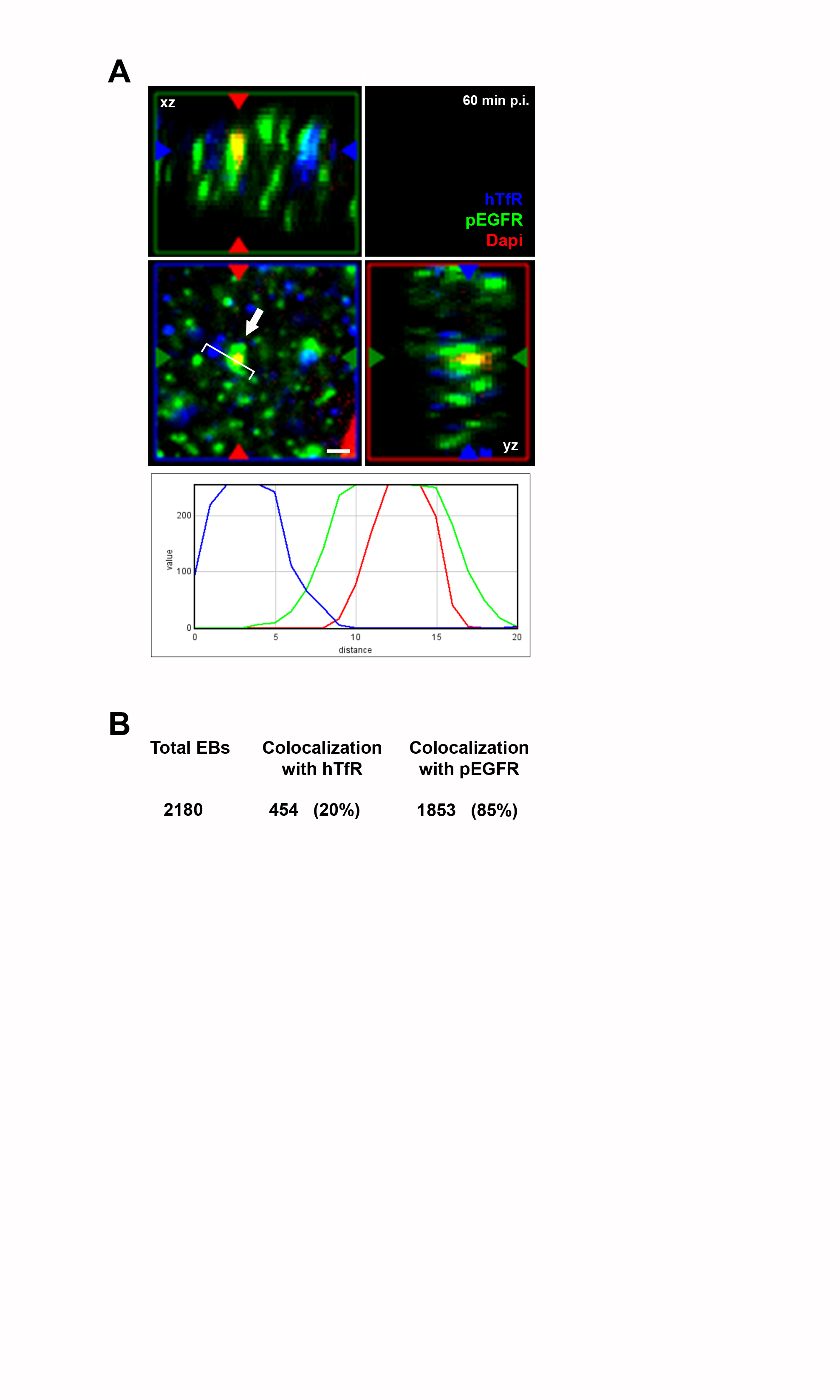

Supplement: Figure S3 — The human transferrin receptor does not colocalize with internalized bacteria (Related to Figures 2C and 5C ). (A) Confocal spinning-disk images of HEp-2 cells infected with C. pneumoniae EBs (MOI 1) for 60 min. Internalized bacteria stained with DAPI (red) are surrounded by activated EGFR, stained with a phospho-EGFR antibody (green). Human transferrin receptor (stained in blue) does not colocalize with the internalized bacteria, as shown in the fluorescence intensity plot (panel below image) generated from a section through the marked area (white arrow). Bar: 1 µm. (B) Quantification of colocalization of EBs with activated EGFR (pEGFR) or human transferrin receptor (hTfR) at 60 min pi. EBs were stained by DAPI, pEGFR and hTfR with specific antibodies. The data represent the means of five independent experiments. (TIF) [file ppat.1003325.s003.tif]

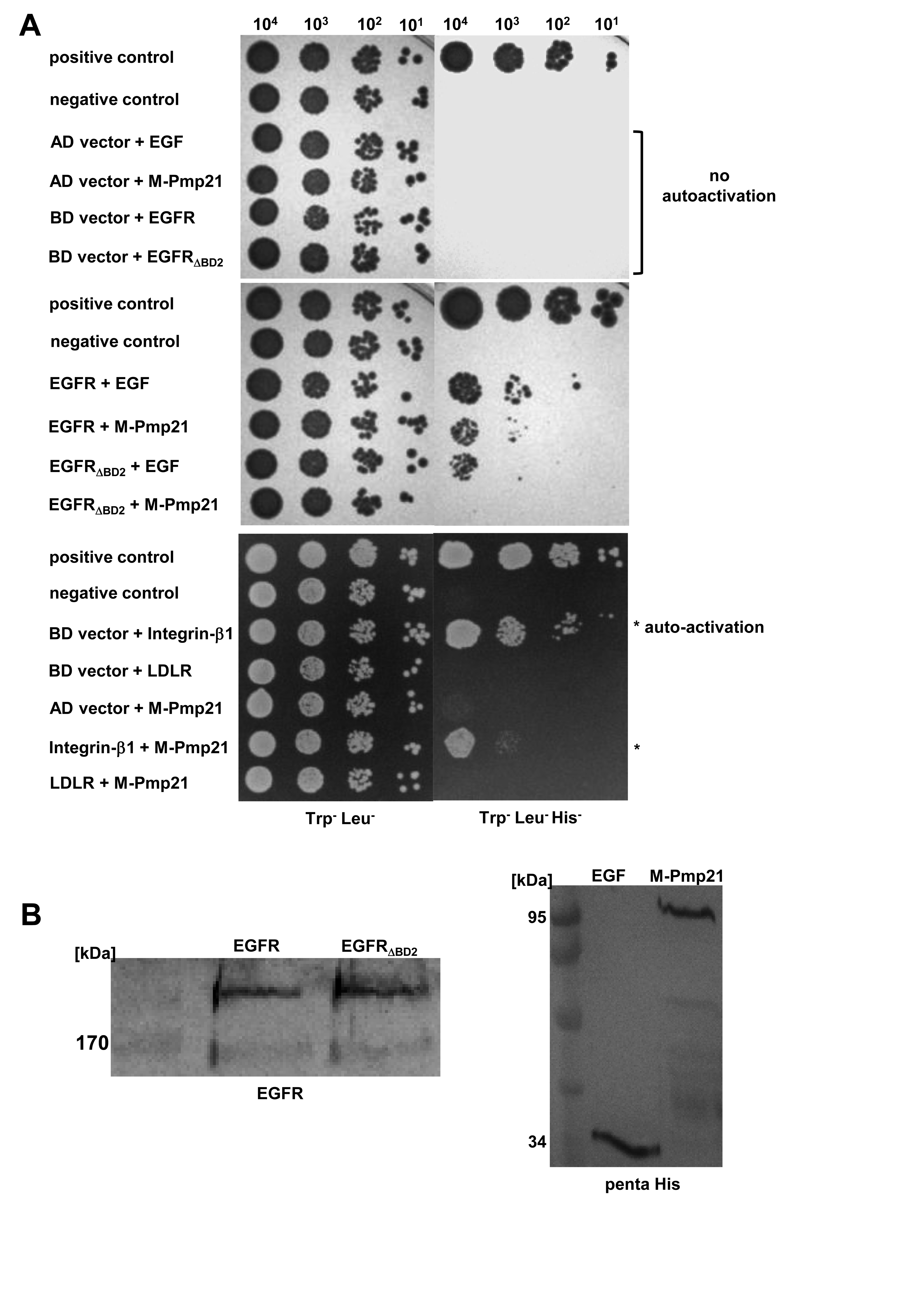

Supplement: Figure S4 — Interaction of EGFR and M-Pmp21 confirmed by yeast two-hybrid analysis (Related to Figure 2E ). (A) Serial dilution patch tests of yeast two-hybrid clones. 104 - 101 yeast cells were patched on selective (Trp−, Leu−) and on low-stringency medium (Trp−, Leu−, His−). The integrin-β1 construct showed autoactivation (*). (B) Immunoblot analysis of yeast cells expressing EGFR or EGFRΔBD2 detected with an EGFR antibody (left). Expression of EGF and M-Pmp21 yeast two-hybrid constructs was detected with a penta-His antibody (right). (TIF) [file ppat.1003325.s004.tif]

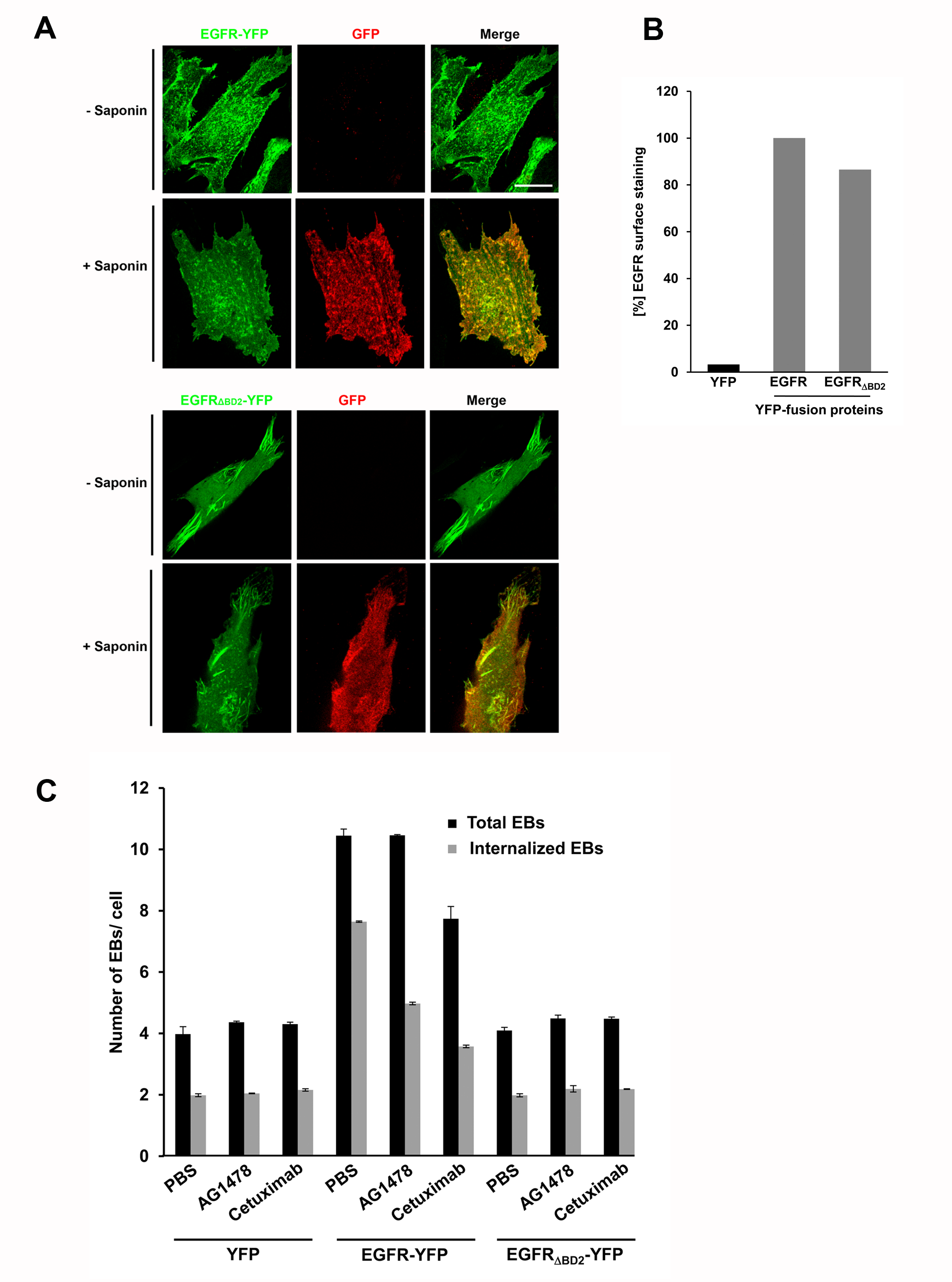

Supplement: Figure S5 — Inhibition of EGFR function in EGFR-expressing CHO-K1 cells reduces rates of EB internalization (Related to Figure 6D ). (A) Confocal spinning-disk images of CHO-K1 cells transfected with EGFR-YFP or EGFRΔBD2-YFP fixed with formaldehyde. Cells were either permeabilized (+ Saponin) or left untreated (− Saponin) and stained with an anti-GFP antibody (red) to detect the C-terminal GFP-tag. Bar: 10 µm. (B) Quantification of the amounts of EGFR-YFP and EGFRΔBD2-YFP expressed on the surface of transfected CHO-K1 cells. Fixed cells were stained first with a mouse antibody that recognizes the EGFR ectodomain (aa 6–273) followed by incubation with FITC-conjugated anti-mouse antibody. Samples of 10,000 transfected CHO-K1 cells each were quantified for cell surface fluorescence by FACS analysis. (C) CHO-K1 cells transfected with EGFR constructs as described in Figure 6A–D were pretreated with the EGFR kinase inhibitor AG1478 or the blocking antibody cetuximab before being exposed to C. pneumoniae EBs The total number of EBs (total) associated with cells and the number of internalized EBs (internalized EBs) were determined as described previously. The data represent the means of four independent experiments. (TIF) [file ppat.1003325.s006.tif]
