# Supplementary material for: The Chlamydia pneumoniae Invasin Protein Pmp21 Recruits the EGF Receptor for Host Cell Entry
Source: PLoS Pathog. 2013 Apr 25;9(4):e1003325. doi: 10.1371/journal.ppat.1003325 (PMC3635982; doi:10.1371/journal.ppat.1003325)
Supplement: Text S1 — Supporting information includes Supplemental Experimental Procedures and a list of relevant Gene Accession Numbers. (DOCX) [file ppat.1003325.s007.docx]

**The *Chlamydia pneumoniae* invasin protein Pmp21 recruits the EGFR receptor for host cell entry**

Katja Mölleken, Elisabeth Becker, Johannes H. Hegemann

**Supplemental Experimental Procedures**

**Biotin pull-down experiments**

Recombinant M-Pmp21 or invasin (200 µg/ml), purified *C. pneumoniae* EBs (5 x 10^8^) and HEp-2 cells (1 x 10^8^) were biotinylated with NHS-SS-biotin, using the Pierce Cell Surface Protein Isolation Kit (Thermo Scientific) according to the manufacturer’s protocol. To identify possible interaction partners, the biotinylated M-Pmp21 (200 µg/ml) was incubated with untreated HEp-2 cells in medium without FCS for 2 h at 37°C. After washing twice with PBS, the cells were exposed, for 30 min at room temperature, to DTSSP (2 mM), which forms cleavable cross-links between the biotinylated M-Pmp21 and nearby cell-surface proteins. The cells were then lysed using Pierce Lysis Buffer. After sonication, the lysate was cleared by a 5-min centrifugation at 13,000 rpm and 4°C. The supernatants were applied to a NeutrAvidin column (Pierce) to capture biotinylated surface protein complexes. After washing the column three times with buffer, bound proteins were eluted with buffer containing 10 mM DTT, which reduces the S-S bond between biotin and protein and cleaves the DTSSP crosslink. Fractions of the eluate were then analyzed by SDS/PAGE. Protein bands identified by Coomassie staining were excised, and subjected to mass spectrometric analysis. Similarly treated lysates of HEp-2 cells that had not been exposed to the biotinylated M-Pmp21 probe served as controls.

To verify the Pmp21-EGFR interaction the same protocol was used, but in this case either *C. pneumoniae* EBs were biotinylated and incubated with non-biotinylated HEp-2 cells or vice versa. Therefore, EBs and human cells were centrifuged for 20min at 2800 rpm and 4°C, before cells were shifted to 37°C for 60min. Then crosslinking and cell lysis was performed as described above. Equal amounts of samples of input and column eluates were analyzed by immunoblotting using antibodies specific for EGFR, M-Pmp21, PDGFR, Integrin-β1, hTfR or Momp.

**Protein expression and affinity purification of His_6_-tagged proteins**

The expression and purification of recombinant His_6_-M-Pmp21 (aa 671-1145), His_6_-invasin (aa 490-986) or His_6_-GST fusion proteins was performed as described previously [1].

**Yeast two-hybrid analyses**

Two-hybrid analyses were carried out using the Matchmaker™ Gold System from Clontech. The pGADT7 vector was altered by inserting a His_6_ tag-coding sequence downstream of the multiple cloning site, resulting in pEB1. Similarly, a VSV tag was integrated into pGBKT7 to generate pEB2. DNA inserts encoding M-Pmp21 or human EGF were cloned into pEB2, while the DNA encoding EGFR (aa 1-1209 according to [2] and the deletion variant EGFR_∆BD2_ were integrated into pEB1. The full length extracellular domains of human LDLR or integrin-β1 were also cloned in pEB1. Interaction of the various constructs was analyzed by serial dilution patch tests on selective (Leu^-^, Trp^-^) and on low-stringency medium (Leu^-^, Trp^-^, His^-^). Expression of M-Pmp21 and EGF was monitored by immunoblot using an anti-VSV antibody (Sigma), expression of EGFR and EGFR_∆BD2_ was analyzed with an EGFR antibody.

**Co-immunoprecipitation of surface complexes from infected cells**

HEp-2 cells (1 x 10^8^) and purified *C. pneumoniae* EBs (MOI 5) were centrifuged for 20 min at 2800 rpm and 4°C. Cells were then shifted to 37°C for 60 min, washed twice with PBS, and DTSSP was added to induce crosslink formation as described above. The membrane-associated EGFR-Pmp21 complex was solubilized by incubation of the cells with 1% n-dodecyl beta-D-maltoside (DDM, Sigma) in PBS for 30 min on ice followed by a 10-sec sonication. The extracts were cleared by centrifugation for 5 min at 13,000 rpm and 4°C, then incubated for 12 hr at 4°C with Aminolink-Sepharose (Thermo Scientific) to which either EGFR, PDGFR or antigen-purified M-Pmp21 antibodies had been covalently coupled. The matrix was washed three times with PBS, and bound proteins were eluted with two 50-µl aliquots of 0.1 M glycine (pH 2.3). The eluted samples and equal amounts of input samples were analyzed by immunoblotting using antibodies raised against EGFR, PDGFR or antigen-purified M-Pmp21.

**Alteration of EGFR expression by transfection procedures**

Depletion of EGFR by specific siRNA was performed by transfection of HeLa229 cells (8 x 10^5^), seeded on glass coverslips, with 80 ng of siRNA per well using Turbofect (Thermo Scientific). Expression of YFP or mCherry-tagged EGFR variants was carried out in CHO-K1 cells (8 x 10^5^), which were transfected with 2 µg of plasmid DNA per well using the CaCl_2_-phosphate transfection method (1 M CaCl_2_, 50 mM BES pH 6.96, 280 mM NaCl, 1.5 mM Na_2_HPO_4_). EGFR expression levels in siRNA-transfected HeLa229 cells and CHO-K1 cells expressing YFP-EGFRs were monitored by immunoblotting. Briefly, cells were lysed 24 h after transfection using 150 µl of Phospho-Lysis buffer per well, proteins were resolved by SDS-PAGE and detected with an EGFR-specific antibody. For quantification of changes in EGFR expression, band intensities were compared using Scion Image software. In CHO-K1 cells the efficiency of transfection was determined (24 h after transfection) by counting the number of fluorescent cells in a sample of approximately 1000 cells using a Zeiss Axioskop.

**Immunofluorescence staining of EBs during early infection**

*C. pneumoniae* EBs (MOI 1) were centrifuged for 20 min at 2800 rpm and 4°C. Cells were then shifted to 37°C. At 5 to 120 min post-infection HEp-2 or transfected CHO-K1 cells on glass coverslips were washed once with TBS, fixed with 3 % formaldehyde for 10 min at room temperature and permeabilized (where necessary) with TBS containing 0.2 % Triton X100 for 10 min. The monolayer was then washed three times with TBS. For detection of the endogenous EGFR, antibodies directed against the C-terminal region, the N-terminal region or against the activated, tyrosine-phosphorylated (Y1068) form were used. Grb2, c-Cbl, or hTfR were stained in combination with phosphor-EGFR. Chlamydial EBs were visualized either with an antigen-purified M-Pmp21 antibody or by staining of DNA with DAPI. All primary antibodies were used together with Alexa594/488-conjugated anti-rabbit or anti-mouse antibodies (Invitrogen). Cells were viewed using a Zeiss Cell Observer® SD confocal spinning-disk microscope (Yokagawa CSU-X1 unit, AxioCam MRm camera). Spinning-disk images were assembled with AxioVision or ZEN2011 software, the 3D reconstitution models were generated with Zeiss ZEN and Image Surfer software, the fluorescence intensity plots by ImageJ software.

The colocalization of chlamydial EBs with phospho-EGFR or hTfR was quantified manually as follows: internalized bacteria were identified by their DAPI signal. Then colocalization of the DAPI signal with the pEGFR signal or the hTfR signal was assessed. Colocalization of bacteria with either receptor was adopted when the receptor signal was overlapping or directly associated with the bacterial DAPI signal. Frequently the pEGFR signal completely or partially surrounded the DAPI signal, while a complete or partial enclosure of the DAPI signal with the hTfR signal was never observed. The colocalization of the adaptor proteins Grb2 and Cbl with bacteria and activated EGFR was done in a very similar way. First internalized bacteria which showed a complete or partial envelope of phospho-EGFR were identified. Then the association or overlap of the pEGFR signal at these bacteria with a Grb2 or Cbl signal was assessed. Importantly, Grb2 or Cbl signals separated from the pEGFR signal by a gap were not considered.

**Internalization of chlamydial particles**

HEp-2 cells, or CHO-K1 cells transfected with plasmids expressing YFP, EGFR-YFP or EGFR_ΔBD2_-YFP, for 24 h were treated with rEGF (100ng/ ml), AG1478 (2µM), cetuximab (5 µg/ ml equals 34nmol/L) or UO126 (10 µM) for 2 h as described above. Cells were then centrifuged for 10 min at 2800 rpm and 4°C in the presence of *C. pneumoniae* EBs (MOI 1), shifted to 37°C for 2 h and then fixed with 3% formaldehyde, without prior permeabilization was performed. External EBs were visualized with antibodies against M-Pmp21 as described above. In five replicate samples of 15 HEp-2 cells per treatment, the number of external EBs was determined and subtracted from the total number of EBs (determined by staining with DAPI) to give the average number of internalized bacteria per cell. Immunofluorescence images were created with the Zeiss Cell Observer® SD spinning-disk confocal microscope.

**Infection experiments**

HeLa229 or CHO-K1 cells transfected with siRNAs or EGFR-YFP constructs were exposed to *C. pneumoniae* GiD (MOI 1) 24 h after transfection. EBs were centrifuged for 10 min at 2800 rpm and 4°C, and then shifted to 37°C for 2 h before infection media was replaced by media containing 1.2 µg/ml cycloheximide. The infection was incubated for 48 h. The number of inclusions formed was quantified by immunofluorescence microscopy, using either a FITC-conjugated antibody directed against chlamydial LPS or antibodies directed against Cpn0147, as described previously.

# **Quantitation of attached *C. pneumoniae* EBs by flow cytometry**

Purified *C. pneumoniae* EBs (2 x 10^8^) were labeled for 1 h at 37°C with 25 µmol of CFSE (Molecular Probes, Invitrogen) and washed twice with PBS containing 1% BSA as previously described [3]. At 24 h after transfection with siRNAs, HeLa229 cells growing in 24-well plates were washed twice with medium, then incubated for 1 h at 37°C with the CFSE-labelled *C. pneumoniae* EBs (MOI 10) without centrifugation. After washing with PBS, cells were detached with Cell Dissociation solution (Sigma) and fixed with 3 % formaldehyde, and the adhesion was measured by flow cytometry using a FACSAria (BD Biosciences).

**Immunoblot detection of phosphorylated and non-phosphorylated proteins**

Confluent monolayers of HEp-2 cells in 24-well plates were starved in serum-free medium for 12 h at 37°C. Cells were kept at 4°C before adding recombinant EGF (100 ng/ml), N/M-Pmp21, M-Pmp21 (100 µg/ml), or viable or non-viable *C. pneumoniae* EBs (MOI 5) without centrifugation and shifting the cells to 37°C. Samples were harvested at 0, 5, 10, 15, 30, 60 min by addition of 150 µl of Phospho-Lysis buffer (1 % NP40, 1 % Triton X100, 20 mM Tris, 140 mM NaCl, 2 mM EDTA, 1 mM Na_2_VO_4_, Protease Inhibitor Cocktail [Roche]) per well. All time points were analyzed by immunoblotting. Membranes were blocked by preincubation with, and antibodies were dissolved in, TBS containing 5 % milk powder. Monoclonal antibodies specific for activated EGFR (pEGFR), EGFR, pERK and β-actin were used and complexes were visualized with AP-conjugated anti-mouse or anti-rabbit antibodies (Promega).

**Cloning procedures**

For the expression of EGFR_∆BD2_ in human cells a deletion of aa 389-438 was generated in the EGFR-YFP construct obtained from Dr. J. Bode. The same deletion was integrated into the two-hybrid EGFR construct. Human LDLR (aa 1-787), integrin-b1 (aa 1-728) or EGF (aa 1-53) were cloned from full length cDNA.

**List of gene accession numbers**

# Homo sapiens Epidermal growth factor receptor (EGFR) NM_005228.3

# Homo sapiens Cbl proto-oncogene, E3 ubiquitin protein ligase (CBL) NM_005188.3

# Homo sapiens Growth factor receptor-bound protein 2 (GRB2) NM_002086.4

# Homo sapiens Platelet-derived growth factor receptor beta (PDGFR) NP_002600.1

# Homo sapiens Human transferrin receptor (hTfR) AAA61153.1

# Homo sapiens Low density lipoprotein receptor (LDLR) NM_000527.4

# Homo sapiens Integrin, beta 1 (CD29) NM_002211.3

Homo sapiens Epidermal growth factor (EGF) [NM_001178131.1](http://www.ncbi.nlm.nih.gov/nucleotide/296011014?report=genbank&log$=nucltop&blast_rank=1&RID=D12DH97K015)

Chlamydophila pneumoniae CWL029 Pmp21 AE001363.1 (1098398-1103227)

Chlamydophila pneumoniae CWL029 GroEL-1 AE001363.1 (167464-169098)

# Y.pseudotuberculosis inv gene encoding invasion M17448.1

**Supplemental References**

1. Moelleken K, Schmidt E, Hegemann JH (2010) Members of the Pmp protein family of Chlamydia pneumoniae mediate adhesion to human cells via short repetitive peptide motifs. Mol Microbiol 78: 1004-1017.

2. Santra M, Reed CC, Iozzo RV (2002) Decorin binds to a narrow region of the epidermal growth factor (EGF) receptor, partially overlapping but distinct from the EGF-binding epitope. J Biol Chem 277: 35671-35681.

3. Schnitger K, Njau F, Wittkop U, Liese A, Kuipers JG, et al. (2007) Staining of Chlamydia trachomatis elementary bodies: a suitable method for identifying infected human monocytes by flow cytometry. J Microbiol Methods 69: 116-121.
